# Disease-specific dynamic biomarkers selected by integrating inflammatory mediators with clinical informatics in ARDS patients with severe pneumonia

**DOI:** 10.1007/s10565-016-9322-4

**Published:** 2016-04-19

**Authors:** Chengshui Chen, Lin Shi, Yuping Li, Xiangdong Wang, Shuanying Yang

**Affiliations:** Department of Respiratory Medicine, The Second Affiliated Hospital of Xi’an Jiaotong University, Xi’an, 710004 China; Department of Pulmonary Medicine, The First affiliated Hospital, Wenzhou Medical University, Wenzhou, 325000 China; Zhongshan Hospital, Shanghai Institute of Clinical Bioinformatics, Fudan University Center for Clinical Bioinformatics, Biomedical Research Center of Fudan University Zhongshan Hospital, Shanghai, China

**Keywords:** ARDS, Biomarkers, Clinical informatics, Proteomics, Pneumonia

## Abstract

Acute respiratory distress syndrome (ARDS) is a heterogeneous syndrome that occurs as a result of various risk factors, including either direct or indirect lung injury, and systemic inflammation triggered also by severe pneumonia (SP). SP-ARDS-associated morbidity and mortality remains high also due to the lack of disease-specific biomarkers. The present study aimed at identifying disease-specific biomarkers in SP or SP-ARDS by integrating proteomic profiles of inflammatory mediators with clinical informatics. Plasma was sampled from the healthy as controls or patients with SP infected with bacteria or infection-associated SP-ARDS on the day of admission, day 3, and day 7. About 15 or 52 cytokines showed significant difference between SP and SP-ARDS patients with controls or 13 between SP-ARDS with SP alone and controls, including bone morphogenetic protein-15 (BMP-15), chemokine (C-X-C motif) ligand 16 (CXCL16), chemokine (C-X-C motif) receptor 3 (CXCR3), interleukin-6 (IL-6), protein NOV homolog (NOV/CCN3), glypican 3, insulin-like growth factor binding protein 4 (IGFBP-4), IL-5, IL-5 R alpha, IL-22 BP, leptin, MIP-1d, and orexin B with a significant correlation with Digital Evaluation Score System (DESS) scores. ARDS patients with overexpressed IL-6, CXCL16, or IGFBP-4 had significantly longer hospital stay and higher incidence of secondary infection. We also found higher levels of those mediators were associated with poor survival rates in patients with lung cancer and involved in the process of the epithelial mesenchymal transition of alveolar epithelial cells. Our preliminary study suggested that integration of proteomic profiles with clinical informatics as part of clinical bioinformatics is important to validate and optimize disease-specific and disease-staged biomarkers.

**Electronic supplementary material:**

The online version of this article (doi:10.1007/s10565-016-9322-4) contains supplementary material, which is available to authorized users.

## Introduction

Adult respiratory distress syndrome (ARDS) is a life-threatening condition manifested as non-cardiogenic pulmonary edema, respiratory distress, and hypoxemia with a high mortality and morbidity in critically ill patients and resulted from various processes that directly or indirectly compromise the lung (Schneider and Sweberg 2013). ARDS was defined as an acute inflammation with neutrophil infiltration and lung epithelial and/or endothelial cell dysfunction, associated with infection (Grommes and Soehnlein 2011). The present therapeutic strategies for ARDS including supportive care, pharmacological treatments, or ventilator support are still controversial, due to the complexity of ARDS and lack of understanding of molecular mechanisms (De Luca et al. 2012). ARDS is commonly caused by sepsis, pneumonia, trauma, aspiration pneumonia, pancreatitis, and other critical clinical conditions (Puneet et al. 2005). Most of patients with severe pulmonary infection become more susceptive to ARDS, have longer duration of hospital stay, or have higher mortality. Infection-associated ARDS is characterized by an uncontrolled inflammatory response to a local or systemic insult, compromising lung alveolar epithelial and endothelial barriers, acute inflammation, edema, or injury. The concomitant clinical course and outcome of ARDS are associated with the degree of systemic inflammation (Lundberg et al. 2000), by which altered production of cytokines and chemokines may occur among different stages and severities of the disease. Clinical and epidemiologic studies have suggested a strong association between chronic infection, inflammation, and cancer (Balkwill and Mantovani 2012). Local infections (e.g., pneumonia and tuberculosis) and inflammation often occur in patients with lung cancer (Engels 2008).

The present study aims at investigating dynamic differences of proteomic profiles between patients with severe pneumonia (SP) or SP accompanied with ARDS (SP-ARDS) on days 1, 3, and 7 after hospital admission, as compared with healthy controls. We have developed the protocol of disease-specific biomarker selection and evaluation by integrating proteomic profiles of inflammatory mediators in pulmonary diseases, e.g., chronic obstructive pulmonary disease, at different stages and durations, with clinical informatics and phenotypes (Chen et al. 2012b). The present study mainly focused on plasma inflammatory mediators measured by antibody microarray, characterized the modes of dynamic alterations in the disease, and selected disease-specific biomarkers by correcting selected biomarkers with Digital Evaluation Score System (DESS) of patients. We also evaluated the potential values of selected biomarkers in the prediction of survival rates, of which higher levels of CXCL6, interleukin-6 (IL-6), or insulin-like growth factor binding protein 4 (IGFBP-4) in the circulation were correlated with poor prognosis in patients with SP-ARDS.

## Materials and methods

### Patient population

Three hundred patients were recruited in the study, of whom 70 patients with bacteria-associated SP-ARDS, with bacteria-associated SP alone, or healthy people as normal controls, separately, were enrolled, as shown in Supplement Table 1. SP was defined by Infectious Disease Society of America/American Thoracic Society (Brown and Dean 2011; Brown et al. 2009). The definition of ARDS was recommended by the Berlin definition in a university hospital (Hernu et al. 2013; Koh 2014; Ranieri et al. 2012), which facilitated easy nomination of patients with ARDS for a randomized, perspective clinical study. Patients with SP or SP-ARDS received corticosteroids, antibiotics, or short-term use of neuromuscular blockade at initial stage of mechanical ventilation and prone ventilation in severe ARDS after blood sample collection for about 7 days when the symptoms were improved (Bellani et al. 2016). All subjects were given informed consent, and the protocol was approved by the Medical Ethics Committee of the First affiliated Hospital, Wenzhou Medical University, China.

### Sampling procedure

Plasma samples were collected intravenously three times from patients with SP or SP-ARDS or healthy controls on the day after the initial diagnosis and the admission (day 1), day 3, and day 7 after the admission and the treatment. The aliquots of plasma were collected in potassium-EDTA tubes, centrifuged at 2000 rpm for 20 min, and then stored at −80 °C until analyses.

### Microarray assay

An antibody-based membrane array for measuring inflammatory factors (A Custom Raybio® Human Inflammation Antibody Array kit) was purchased from Raybiotech (Norcross, GA, USA) with 507 selected inflammatory mediators. Each antibody was spotted in duplicate onto one membrane. The antibody microarray consists of numerous affinity reagents arrayed on a solid surface, and proteins that bind specific target proteins to unique locations on the array are subsequently detected (MacBeath 2002). Briefly, membranes immobilized with capture antibodies were blocked with 5 % bovine serum albumin/Tris-buffered saline (TBS) for 1 h and then incubated with 1 ml samples in tenfold dilution with 5 % bovine serum albumin/TBS for 2 h at room temperature. Membranes were then incubated individually or collectively with biotin-conjugated antibodies, after extensive washes with TBS/0.1 % Tween 20 and TBS thrice to remove unbound proteins. Membranes were then incubated with horseradish peroxidase-conjugated streptavidin at 2.5 pg/ml for 1 h at room temperature. Unbound materials were washed out with TBS/0.1 % Tween 20 and TBS. Buffers C and D were then mixed and loaded onto the membranes to cover the entire surface for 5 min. Finally, spots were detected by exposing to Kodak X-Omat radiographic film for 1 min for image. Each film was scanned with Scanalyze software, and spots were digitized into densities. The data were exported into Microsoft Excel, and for each spot, the net density was determined by subtracting the background density from the total raw density. The relative spot density in each membrane equals to [the average of inflammatory mediator spot density − blank density] / [the average of positive control density − blank density] × 100 %. According to the protocol from the manufacturer, the positive signals are used to identify the orientation and compare the relative expression levels among the different membranes. Horseradish peroxidase-conjugated antibody served as a positive control as 12 spots and was also used to identify the orientation. The positive control of density in the microarray was the known protein in the controlled concentration to control the quality of sample load, the density of spots, and the operation.

### DESS

DESS is a score index to translate clinical descriptions and information into clinical informatics, which took into account patient symptoms, signs, doctor examination, biochemical analyses, and clinical imaging, as described previously (Chen et al. 2012a). Variables in the DESS included symptoms in Supplement Table 2, signs in Supplement Table 3, and clinical biochemical analysis in Supplement Table 4. For the assessment of severity, each component was then assigned with 0, 1, 2, and 4 as shown in Supplement Tables 2–4. The score of 4 as the maximal value indicates far more above normal range or much severer condition, while 0 as the minimal value indicates that it is within physiological range. Several variables were 0 or 4, e.g., orthopnea at night, chill, three depression signs, barrel chest, etc. The value of 3 was missed in the scoring system for exponential values in order to better define the severity stages. After compiling patients’ data, the points of each variable were added so that the DESS scores ranged from 0 to 264 points, with higher scores indicating a severer condition. Patients were scored on the day when plasma samples were collected.

### Gene function analyses

All genes correspondent to measured proteins were enrolled in further bioinformatic analyses. We used GenCLip 2.0 (http://ci.smu.edu.cn/GenCLiP2.0/analysis.php) for gene cluster analysis (Huang et al. 2008) and Molecule Annotation System (MAS 3.0) (http://bioinfo.capitalbio.com/mas3/) to generate gene ontology (GO) gene function annotation.

### Survival analyses

Mediators, measured in patients with SP-ARDS and demonstrated statistically significant as compared with either those with SP alone or healthy controls, were furthermore assayed in plasma and the correlation with the survival rate in additional patients with diseases from the hospital was evaluated (*n* = 300). Genes of selected specific proteins in patients with SP-ARDS and correlated with the survival rate were further analyzed for survival prediction values for patients with lung cancer. Univariate associations between expression profiles and survivals were assessed by Cox regression using the coxph function from the R statistical software package “survival.” Differences between survival curves and log rank *P* values were assessed using the survdiff function of the “survival” package. The normalized RNA-seq data from 528 ADC samples and 532 SCC samples were obtained from Broad GDAC FIREHOSE on 7 November 2013. In addition, the clinical survival data were obtained from the Cancer Genome Atlas (TCGA) FTP server (https://tcga-data.nci.nih.gov/tcgafiles/ftp_auth/distro_ftpusers/anonymous/tumour) on 6 December 2013. These data were used to build survival models. Besides, another online survival prediction database including 1715 non-small cell lung cancer samples of ten independent datasets was also used to build survival models (Gyorffy et al. 2013).

### Epithelial mesenchymal transition (EMT) measurement

A549 cells were cultured with 5, 50 , or 500 ng/ml IL-6 in six-well plate (×105 cells/well) for 48 h. Total RNA was isolated using a guanidinium isothiocyanate/chloroform-based technique (TRIzol, Invitrogen, USA) and measured with OD 260 nm. RNA was subsequently reversed and transcribed to cDNA with the SuperScript First-Strand Synthesis System (Invitrogen, USA). Quantitative RT-PCR was carried out using an ABI 7000 PCR instrument (Eppendorf, Hamburg, Germany) with the two-stage program parameters, as follows: 1 min at 95 °C and then 40 cycles of 5 s at 95 °C and 30 s at 60 °C. The sequences of the primer sets used for this analysis are as follows: E-cadherin, 5′-CCCACCACGTACAAGGGTC-3′ (forward [F]) and 5′-CTGGGGTATTGGGGGCATC-3′ (reverse [R]); vimentin, 5′-CGCCAGATGCGTGAAATGG-3′ (F) and 5′-ACCAGAGGGAGTGAATCCAGA-3′ (R); and for human glyceraldehyde-3-phosphate dehydrogenase (GAPDH), 5′-CCACCCATGGCAAATTCCATGGCA-3′ (F) and 5′-TCTACACGGCAGGTCAGGTCCACC-3′ (R). Specificity of the produced amplification product was confirmed by examination of dissociation reaction plots. Each sample was tested in triplicate with quantitative RT-PCR, and each group had six wells.

### Western blot analysis

Intracellular protein was extracted by radio immunoprecipitation assay lysis buffer 48 h after IL-6 stimulation. Protein samples (50 μg) were mixed with an equal volume of 5× sodium dodecyl sulfate buffer, boiled for 5 min, and then separated through 10 % sodium dodecyl sulfate-polyacrylamide gel electrophoresis gels. After electrophoresis, proteins were transferred to polyvinylidene fluoride membranes by electrophoretic transfer. Membranes were blocked in 5 % dry milk for 2 h, rinsed, and incubated with primary antibodies (diluted at their instructions) in TBS thrice at 4 °C overnight. Primary antibody was then removed by washing in TBS and labeled by incubating with 0.1 mg/ml peroxidase-labeled secondary antibodies (against mouse and rabbit) for 2 h. Following three washes in TBS, bands were visualized by ECL and exposed to X-ray film. All results were calculated by Phoretix 1D software.

### Statistical analysis

Statistical analysis were performed by SPSS software (SPSS 18.0; SPSS Inc., Chicago, IL). Signal densities of microarrays among the three groups were analyzed with one-way ANOVA, followed by an unpaired Student’s *t* test to compare the difference between two groups, when the ANOVA test indicated significance. The subset of mediators with significance among groups was then selected. Correlation analysis between total DESS and selected mediator intensities was performed by the nonparametric Spearman correlation test. All data were expressed as mean ± SEM, and a *P* value of <0.05 was considered statistically significant.

## Results

Thirty-six DESS variables of patients with SP-ARDS on day 1 were significantly higher than those on days 3 and 7 as listed in Table 1, and 28 variables in patients with SP on day 3 were significantly higher than those on days 1 and 7 (Table 1). DESS scores represented the severity of patients and declined as the condition improved. Total DESS values in SP-ARDS patients were 544, 339, or 285 on post-admission 1, 3, or 7 days, respectively, and DESS values on day 1 were significantly higher than on days 3 and 7 (Table 2; *P* < 0.01, respectively). While total DESS values in SP patients were 274, 410, or 250 on post-admission 1, 3, or 7 days, respectively, and DESS values on day 3 were significantly higher than days 1 and 7 (Table 3; *P* < 0.05, respectively).

**Table 1:** Variables for seven ALI/ARDS and five severe pneumonia (SP) patients on days 1, 3, and 7 (mean ± SEM)

Variables	Day 1		Day 3		Day 7	
	ARDS	SP	ARDS	SP	ARDS	SP
Cough severity	0.857 ± 0.34	1.17 ± 0.43	0	3.21 ± 0.691	0	1.211 ± 0.632
Sputum	2.286 ± 0.644	1.572 ± 0.584	1.429 ± 0.683	3.337 ± 0.884	1.286 ± 0.583	1.299 ± 0.631
Shortness of breath	2.714 ± 0.474	0.564 ± 0.347	1.286 ± 0.36	1.286 ± 0.36	0.857 ± 0.404	0
Limitation of activity	3.429 ± 0.369	0	2.143 ± 0.553	0.932 ± 0.411	2 ± 0.577	0
Chill	2.286 ± 0.808	0	0	1.043 ± 0.436	0	0
Fever (°C)	1.714 ± 0.644	1.378 ± 0.524	0.857 ± 0.143	1.921 ± 0.684	0	0
Appetite	2.143 ± 0.705	0.843 ± 0.662	1 ± 0.378	1.893 ± 0.618	0.714 ± 0.286	0.997 ± 0.583
Stool and urine	1.143 ± 0.738	0	0	1.214 ± 0.594	0	0
Temperature (°C)	2.286 ± 0.474	1.686 ± 0.872	1 ± 0.378	2.495 ± 0.773	0.286 ± 0.184	1.186 ± 0.564
Heart rate (beat/min)	3.429 ± 0.571	1.413 ± 0.654	1.714 ± 0.808	1.714 ± 0.808	1.714 ± 0.808	0
Respiratory rate (min)	2.571 ± 0.528	1.267 ± 0.634	1.143 ± 0.595	1.943 ± 0.664	0.857 ± 0.34	0
Blood pressure (mmHg)	1 ± 0.309	0	0.286 ± 0.184	0.763 ± 0.127	0.143 ± 0.143	0
Barrel chest	1.714 ± 0.808		0.571 ± 0.571		0.571 ± 0.571	
Chest palpitation	1.714 ± 0.808	0.736 ± 0.408	0	1.694 ± 0.512	0	0
Chest percussion	3.429 ± 0.571	0	1.143 ± 0.738	0.971 ± 0.369	1.333 ± 0.843	0
Rales	2 ± 0.577	1.774 ± 0.5769	1 ± 0.378	3.125 ± 0.878	0.714 ± 0.36	1.462 ± 0.579
WBC (×10^9^/L)	2.857 ± 0.738	1.818 ± 0.697	1.571 ± 0.481	2.171 ± 0.583	1.143 ± 0.508	1.512 ± 0.508
Neutrophil percentage (%)	3.429 ± 0.571	1.729 ± 0.851	1.629 ± 0.528	2.743 ± 0.828	1 ± 0.535	1.242 ± 0.535
Albumin (g/L)	2.571 ± 0.469		1.143 ± 0.595		1.714 ± 0.68	
ALT (U/L)	2.286 ± 0.606		1.143 ± 0.738		0.571 ± 0.571	
AST (U/L)	2.036 ± 0.714		1.286 ± 0.521		1.132 ± 0.734	
ALP (U/L)	2.286 ± 0.606		1.143 ± 0.738		0.571 ± 0.571	
Urea (mmol/L)	2.857 ± 0.595	0	1.286 ± 0.644	1.166 ± 0.574	1 ± 0.378	0
HDL (mmol/L)	1.429 ± 0.685		0.143 ± 0.143		0	
Na (mmol/L)	0.429 ± 0.202		0.143 ± 0.143	0.843 ± 0.137	0	
K (mmol/L)	1.857 ± 0.769	1.186 ± 0.508	0.286 ± 0.184	1.798 ± 0.614	0.286 ± 0.184	0
Cl (mmol/L)	2.286 ± 0.808		0.571 ± 0.571		0.571 ± 0.571	
Ca (mmol/L)	0.571 ± 1.512		0		0	
pH	1.714 ± 1.808	1.623 ± 0.708	0.571 ± 0.571	1.771 ± 0.614	0.571 ± 0.571	0.571 ± 0.571
PaO_2_ (mmHg)	1 ± 0.218	1.312 ± 0.514	0.429 ± 0.202	2.129 ± 0773	0.286 ± 0.184	1.486 ± 0.594
PaCO_2_ (mmHg)	2.859 ± 0.952	1.783 ± 0.652	1.383 ± 0.718	2.113 ± 0.917	1.714 ± 0.808	1.524 ± 0.714
SaO_2_ (%)	1.571 ± 0.218	1.157 ± 0.418	0.829 ± 0.534	1.932 ± 0.589	0.429 ± 0.535	0.919 ± 0.417
C-reactive protein, CRP (mg/L)	3.143 ± 0.404	2.243 ± 0.804	1.286 ± 0.565	1.986 ± 0.992	0.857 ± 0.459	0.857 ± 0.338
Lung consolidation	2.167 ± 0.654	1.476 ± 0.558	0.833 ± 0.307	1.833 ± 0.344	0.5 ± 0.224	1.105 ± 0.667
Pleural effusion	1.5 ± 0.563	1.605 ± 0.821	0.756 ± 0.27	1.997 ± 0.693	0.333 ± 0.221	1.023 ± 0.775
Emphysema	2.667 ± 0.843	1.787 ± 0.615	1.333 ± 0.769	1.923 ± 0.801	0.667 ± 0.667	1.206 ± 0.657

**Table 2:** Scores of seven ALI/ARDS patients on each day when samples were collected

	ALI/ARDS-1	ALI/ARDS-3	ALI/ARDS-7
Patient 1	74	35	57
Patient 2	57	42	19
Patient 3	76	47	50
Patient 4	103	75	60
Patient 5	82	26	31
Patient 6	77	54	27
Patient 7	75	60	41
Total scores	544	339	285
Mean	77.71428571	48.42857143	40.71428571
SEM	5.144179724	6.167723756	5.931009252
No,	7	7	7
	*P*	0.001760105	0.000265417
		*P*	0.192531293

**Table 3:** Scores of five severe pneumonia patients on each day when samples were collected

	Severe pneumonia 1	Severe pneumonia 3	Severe pneumonia 7
Patient 1	24	61	37
Patient 2	55	100	38
Patient 3	91	94	81
Patient 4	60	85	74
Patient 5	44	70	20
Total scores	274	410	250
Mean	54.8	82	50
SEM	10.96083938	7.286974681	11.7260394
No.	5	5	5
	*P*	0.038926513	0.386280889
		*P*	0.027623376

Levels of inflammatory mediators in patients with SP were significantly altered on day 3, as compared with controls, consistent with the DESS scores as shown in Table 3. SP patients had similar alterations of inflammation-associated proteins, e.g., insulin-like growth factor I receptor (IGF-I sR), insulin-like growth factor II (IGF-II), lipopolysaccharide-binding protein (LBP), or leukocyte cell-derived chemotaxin 2 (LECT2), to SP-ARDS patients. C-C motif ligand 21 (6Ckine), lipopolysaccharide receptor (CD14), interleukin-1 receptor 4 (IL-1 R4/ST2), insulin-like growth factor binding protein 2 (IGFBP-2), insulin-like growth factor I (IGF-I), and defensin-beta1 (BD-1) were significantly changed in SP patients, as compared with healthy controls or SP-ARDS patients at corresponding days.

Thirteen inflammatory mediators in SP-ARDS patients showed significant difference from healthy controls or SP patients, including bone morphogenetic protein-15 (BMP-15), chemokine (C-X-C motif) ligand 16 (CXCL16), chemokine (C-X-C motif) receptor 3 (CXCR3), IL-6, protein NOV homolog (NOV/CCN3), glypican 3, IGFBP-4, IL-5, IL-5 receptor alpha (IL-5Rα), IL-22 binding protein (IL-22BP), leptin, macrophage inflammatory protein-1d (MIP-1d), and orexin B. Among them, clinical informatics, such as symptoms, signs, laboratory tests, and imaging, had significant correlation with those 13 different proteins listed in Supplement Tables 5–8.

**Fig. 1:**
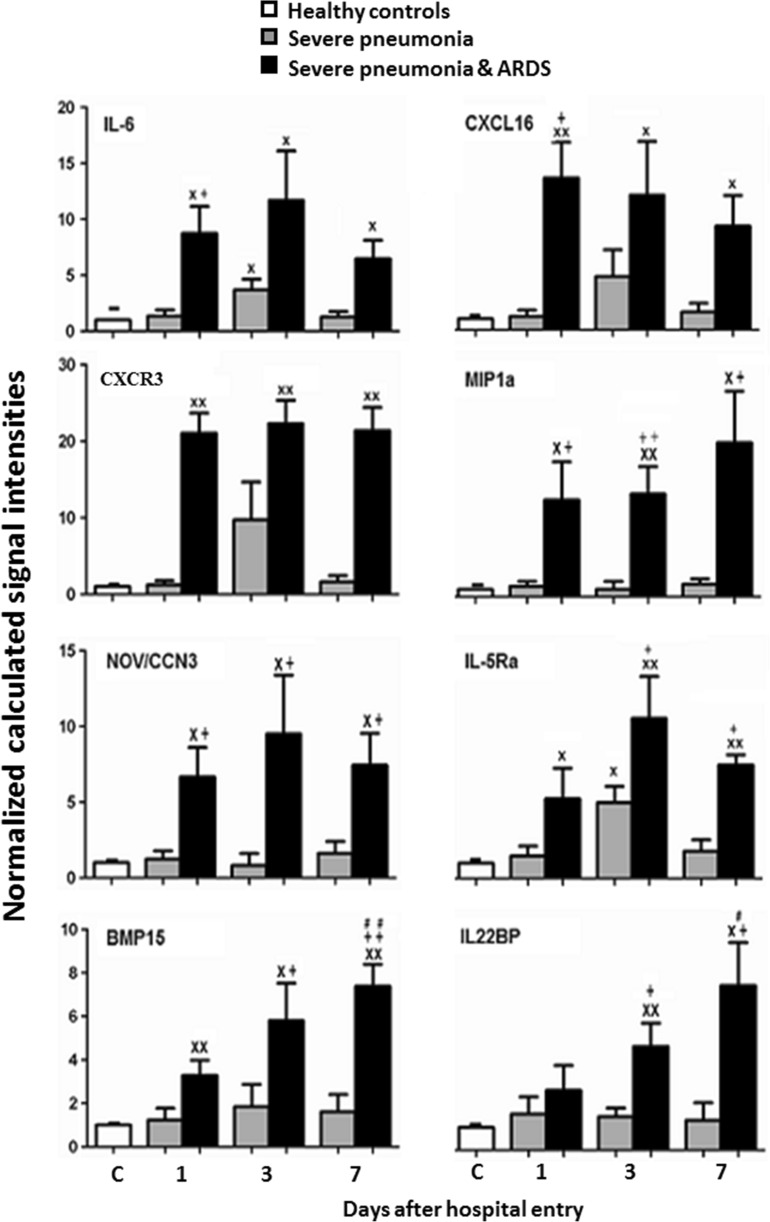
Plasma levels of IL-6, CXCL16, CXCR3, MIP-1d, NOV/CCN3, IL-5 R alpha, BMP-15, and IL-22BP in healthy and patients with SP or ARDS on days 1, 3, and 7. *Single letter x* and *double letter x* stand for *P* values less than 0.05 and 0.01, respectively, as compared with healthy controls. *Single plus sign* and *double plus sign* stand for *P* values less than 0.05 and 0.01, respectively, as compared with SP patients. *Single number sign* and *double number sign* stand for *P* values less than 0.05 and 0.01, as compared with ARDS patients on day 1

**Fig. 2:**
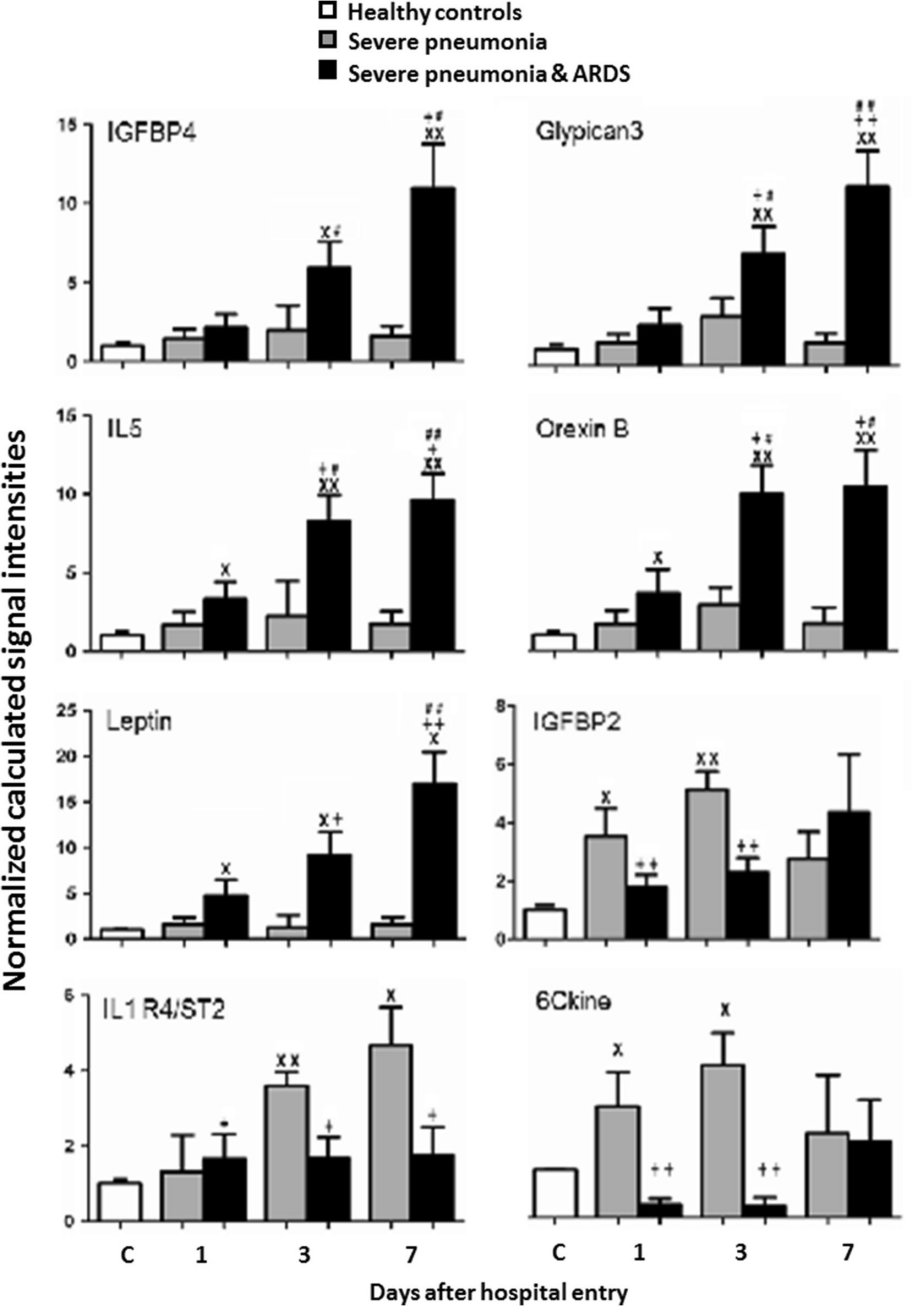
Plasma levels of IGFBP-4, glypican 3, IL-5, leptin (OB), orexin B, IGFBP-2, IL-1 R4/ST2, and 6Ckine in healthy and patients with SP or ARDS on days 1, 3, and 7. *Single letter x* and *double letter x* stand for *P* values less than 0.05 and 0.01, respectively, as compared with healthy controls. *Single plus sign* and *double plus sign* stand for *P* values less than 0.05 and 0.01, respectively, as compared with SP patients. *Single number sign* and *double number sign* stand for *P* values less than 0.05 and 0.01, as compared with ARDS patients on day 1

**Fig. 3:**
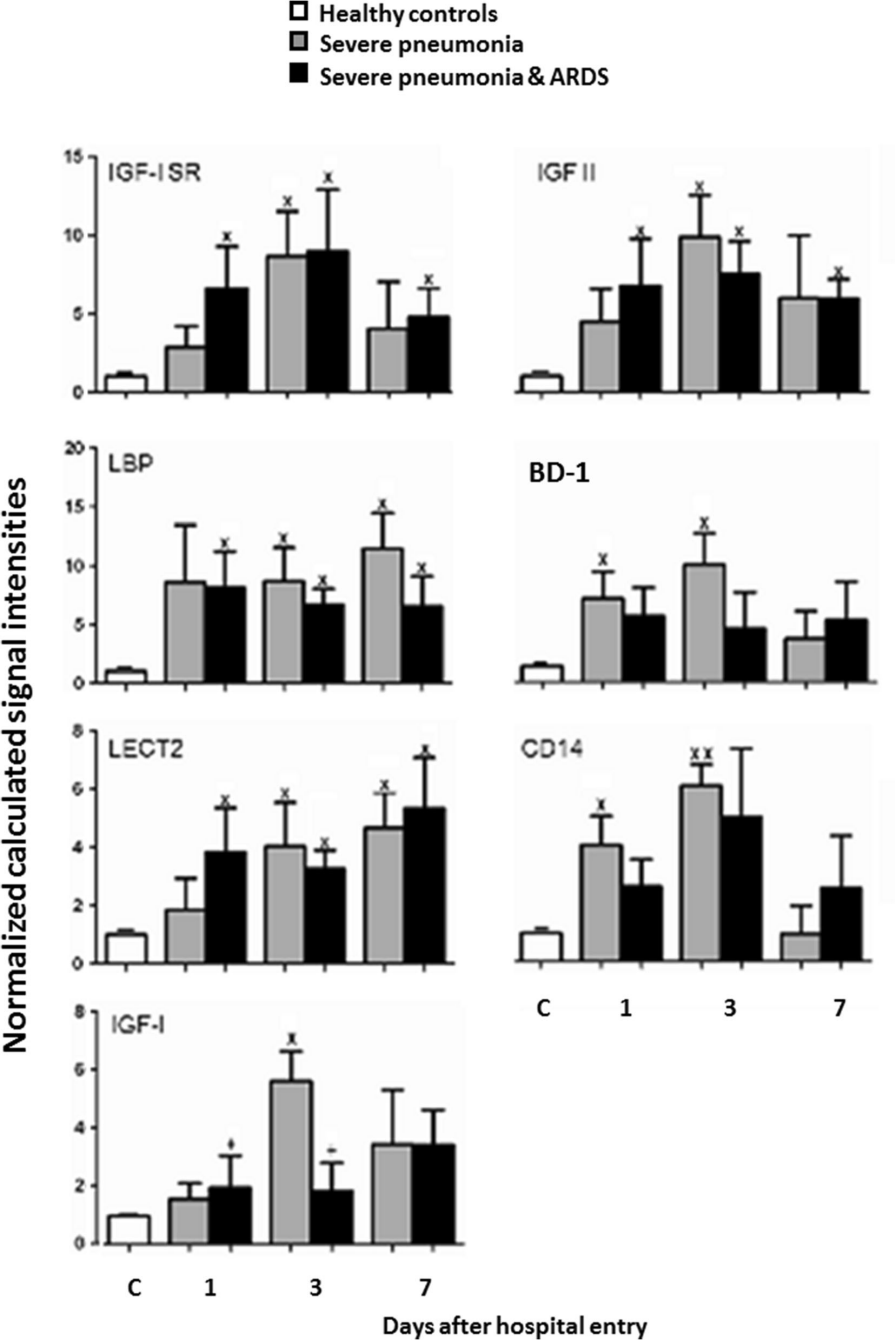
Plasma levels of IGF-I sR, IGF-II, LBP, BD-1, LECT2, CD14, and IGF-I in healthy and patients with SP or ARDS on days 1, 3, and 7. *Single letter x* and *double letter x* stand for *P* values less than 0.05 and 0.01, respectively, as compared with healthy controls. *Single plus sign* and *double plus sign* stand for *P* values less than 0.05 and 0.01, respectively, as compared with SP patients

Levels of IL-6, IL-5Rα, CXCR3, or CXCL16 in patients with SP-ARDS on day 3 were significantly higher than those in SP patients or controls and in SP alone higher than in controls (Fig. 1, *P* < 0.05 or 0.01, respectively). Levels of IL-6, IL-5Rα, CXCR3, CXCL16, NOV/CCN3, MIP-1d, or BMP-15 in patients with SP-ARDS increased significantly from day 1 and on and remained significantly higher during the hospital stay, as compared with both SP alone or controls. Levels of BMP-15, IGFBP-4, glypican 3, IL-22BP, IL-5, leptin, or orexin B in SP-ARDS patients gradually and significantly increased by time as compared with the previous day and from day 1 or day 3 and on as compared with controls or SP alone (Figs. 1 and 2, *P* < 0.05 or less, respectively). Levels of 6Ckine and IGFBP-2 or IL-1R4/ST2 in SP patients were significantly higher from day 1 or day 3 and on as compared with those in controls and in SP-ARDS patients, as shown in Fig. 2 (*P* < 0.05 or less). Figure 3 demonstrated that levels of IGF-I sR, IGF-II, LBP, LECT2, CD14, IGF-I, or BD-1 increased in both SP and SP-ARDS patients from day 1 and on, as compared with controls.

**Fig. 4:**
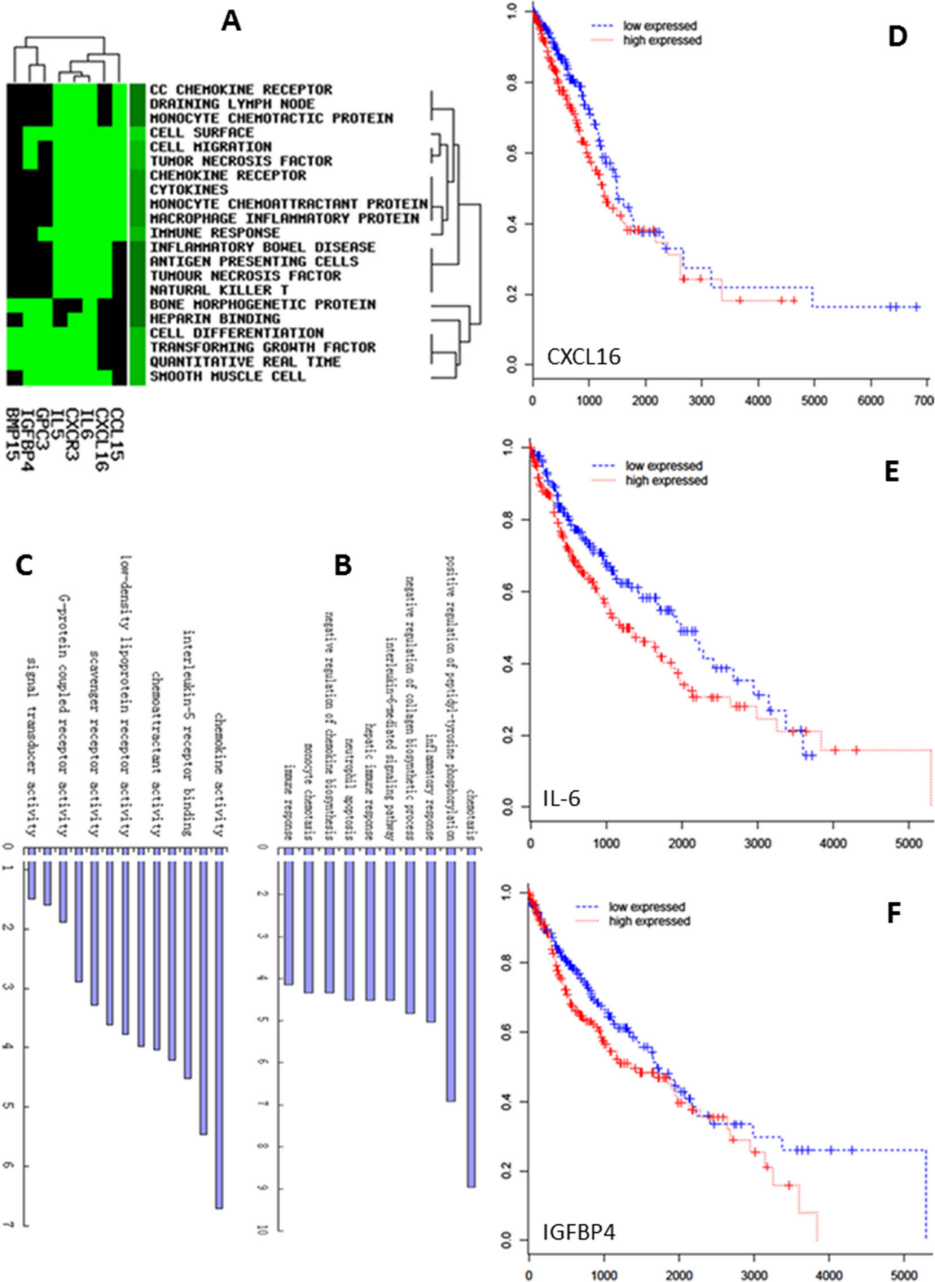
The alterations of gene clusters (**a**), biological processes (**b**), and molecular function (**c**) in ARDS patients. The poor overall survival rate in patients with lung cancer predicted by selected genes CXCL16 (**d**), IL-6 (**e**), and IGFBP-4 (**f**)

Gene clusters associated with CC chemokine receptors, draining lymph nodes, monocyte chemotactic proteins, cell surfaces, cell migrations, cell differentiations, tumor necrosis factor, or immune responses significantly up-expressed in SP-ARDS patients (Fig. 4a). Ten most frequent GO terms were selected for GO biological process and molecular function analysis in the present study. The GO biological processes of up-expressed genes mainly included immune response, monocyte chemotaxis, negative regulation of chemokine biosynthesis, neutrophil apoptosis, hepatic immune response, IL-6-mediated signaling pathway, negative regulation of collagen biosynthetic process, inflammatory response, positive regulation of peptidyl-tyrosine phosphorylation, and chemotaxis in SP-ARDS patients (Fig. 4b). In GO molecular function, genes related with signal transducer activity, receptor activity, G protein-coupled receptor activity, heparin binding, scavenger receptor activity, chemokine receptor activity, low-density lipoprotein receptor activity, C-X-C chemokine receptor activity, chemoattractant activity, IL-6 receptor binding, IL-5 receptor binding, cytokine activity, and chemokine activity up-expressed in SP-ARDS patients (Fig. 4c).

**Fig. 5:**
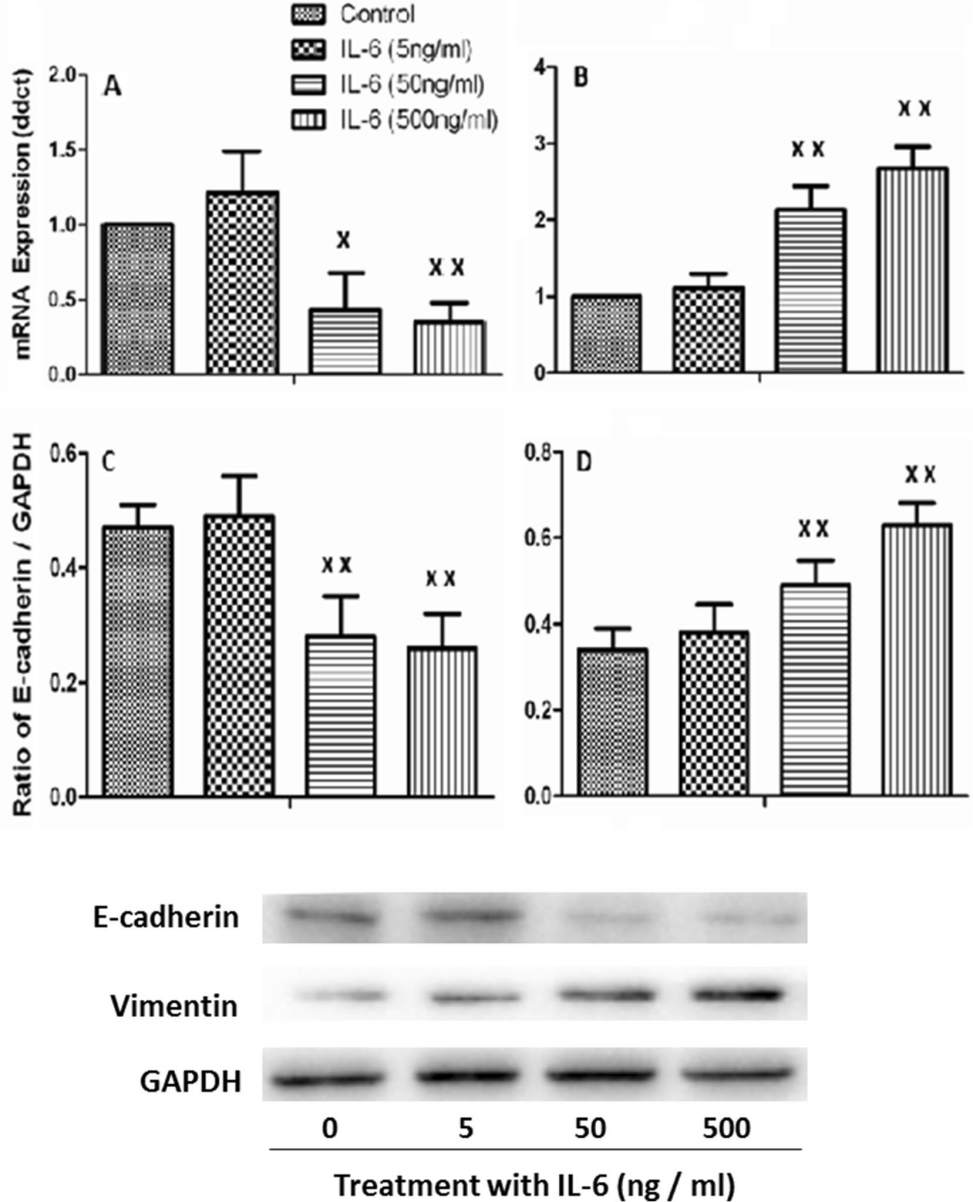
IL-6 promotes a gene expression pattern and phenotype consistent with EMT. A549 cells were treated with 5, 50, or 500 ng/ml IL-6 for 24 h. Real-time quantitative PCR analysis of A549 cells showed a robust decrease in E-cadherin gene expression (**a**) and concomitant increase in vimentin (**b**). Western blot analysis of A549 showed the expressions of E-cadherin were significantly decreased (**c**) and vimentin was up-regulated (**d**) after 48 h stimulated by IL-6. Each data point represents mean ± SEM of three experiments. *Single letter x* and *double letter x* stand for *P* values less than 0.05 and 0.01, in comparison with untreated control cells

We further measured mRNA expression of selected 13 ARDS-specific inflammatory mediators in circulating leukocytes of 200 patients with ARDS and found that ARDS patients with higher expression of IL-6, CXCL16, or IGFBP-4 had longer hospital stay (18.6 ± 4.3 vs 9.3 ± 3.2 days) and higher incidence of secondary infection (36.5 ± 6.2 vs 12.4 ± 3.6 %), respectively. We also found that the expression of IL-6, CXCL16, or IGFBP-4 significantly predicted the poor overall survival in patients with lung cancer (Fig. 4d–f). Of those, IL-6 was one of the strongest poor prognosis predictors. Furthermore, we found higher levels of those mediators were involved in the process of the EMT of alveolar epithelial cells. To characterize whether IL-6 is capable of inducing an EMT phenotype to promote lung cancer, we investigated the gene and protein expression of the epithelial marker E-cadherin and the mesenchymal marker vimentin and found an expression pattern of E-cadherin repression (Fig. 5a) and concomitant induction of vimentin (Fig. 5b) in lung cancer cells as compared with control cells. To further study the effects of IL-6 on A549 cells, we detected EMT phenotype protein by western blot and found that the expression of E-cadherin was significantly decreased 48 h after IL-6 administration at 50 or 500 ng/ml (Fig. 5c). The expression of vimentin significantly increased with IL-6 at 50 ng/ml for 48 h in A549 cells and even more significantly increased at 500 ng/ml (Fig. 5d).

## Discussion

Inflammation plays an important role in the pathogenesis of ARDS, a severe form of acute lung injury, characterized by the activation of leukocytes, dysfunction of the endothelial and epithelial barrier, leakage of a protein-rich exudate from the circulation to the alveolar space and interstitial tissues, or lung injury and disability of gas exchange (Shields et al. 2002). Overactivated leukocytes, endothelial cells, and/or epithelial cells could produce and release a large number of inflammatory mediators, responsible for the initiation and acceleration of the secondary inflammatory reactions (Wang et al. 2007). A panel of altered systemic biomarkers may provide more and deeper understanding of the pathogenesis, monitor the development of the disease and responses to therapies, and identify optimal therapies or personalized medicine, since the single mediator could not reflect the complex of the inflammation in ARDS.

**Fig. 6:**
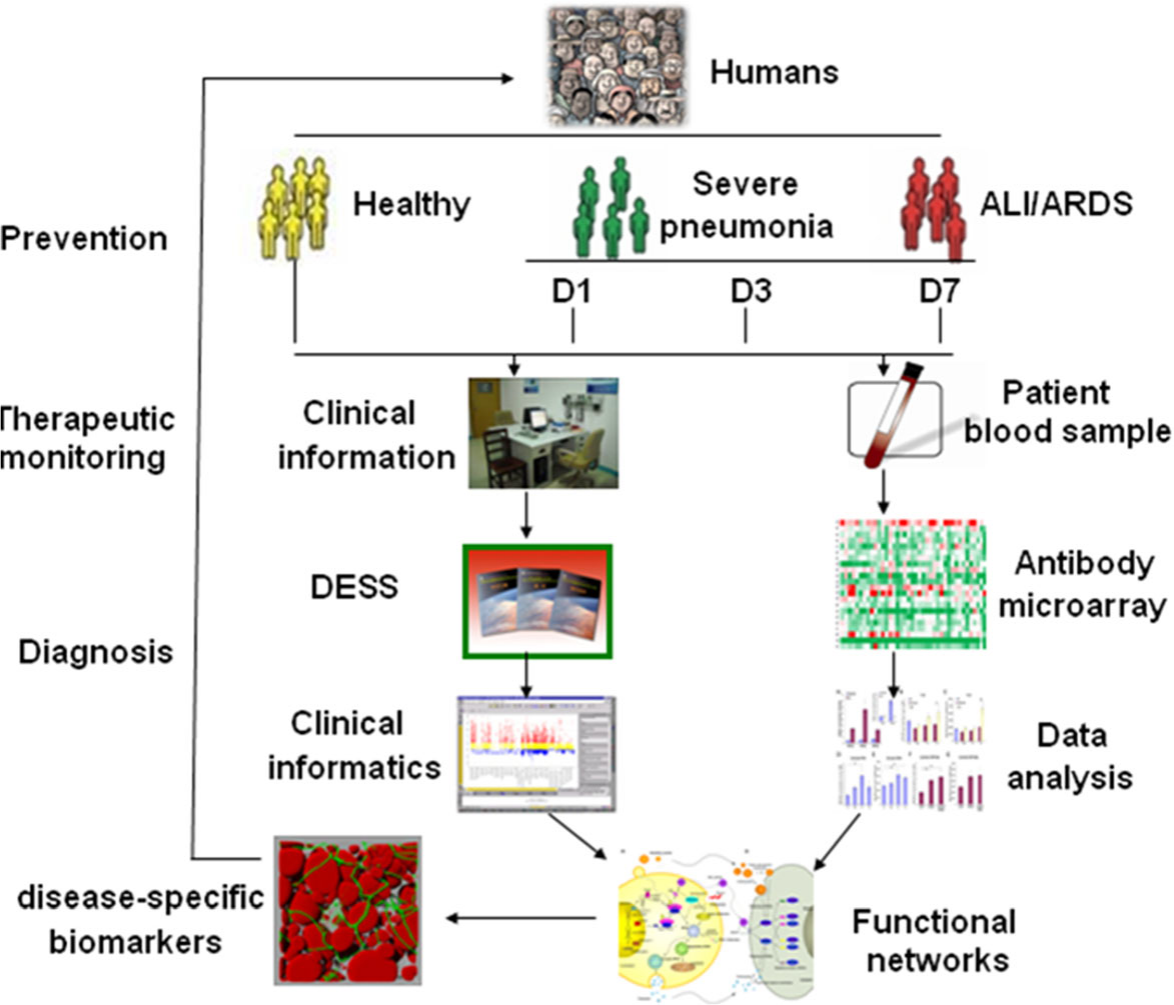
Workflow of the present study. As a new protocol of biomarker evaluation and development, it is achieved by comparing systemic profiles of inflammatory mediators among different study groups, integrating clinical informatics and bioinformatics, and understanding the biological function and signal networks. Clinical informatics is generated through a new DESS, while circulating inflammatory mediators are measured by the antibody array and followed by proteomic-based bioinformatics. Disease-specific biomarkers are identified by integrating clinical informatics and functional networks through the global proteomic data set, in order to develop preventive, diagnostic, and predictive methods for personalized medicine

The present study explored systemic profiles of inflammatory mediators among SP patients with or without ARDS at difficult disease stages and severities by integrating clinical informatics and bioinformatics and understanding the biological function and signal networks, in order to identify the disease-specific biomarkers and develop preventive, diagnostic, and predictive methods for personalized medicine as summarized in Fig. 6. It was highly recommend that validated biomarkers based on clinical proteomics should be integrated with medical imaging with clinical care, personalized treatment paradigms to reduce mortality, and healthcare costs of the diseases (Kato et al. 2011). An integrative systems biology research strategy could overcome limitations in identifying functional and regulatory pathways (Auffray et al. 2010) and predicting multi-scale models ranging from the molecule to the organ levels. The present study investigated networks of selected ARDS-specific inflammatory mediators, e.g., BMP-15, CXCL16, CXCR3, IL-6, NOV/CCN3, glypican 3, IGFBP-4, IL-5, IL-5 R alpha, IL-22 BP, leptin, MIP-1d, or orexin B, and suggested disease-specific biomarkers as explained in Supplement Figs. 1–11.

Infection-induced SP is one of common and critical pathogeneses in the development of ARDS (Soma 2013). The present study was designed to identify 13 dynamic biomarkers of SP-ARDS, different from the healthy or SP patients alone. The severity of the disease was reflected by variations of BMP-15, glypican 3, IGFBP-4, IL-5, IL-22 BP, leptin, or orexin B between day 1 and day 7 of ARDS patients, while the representative of SP was defined by the differentiation from the healthy and ARDS patients (e.g., 6Ckine, CD14, IL-1 R4 /ST2, IGFBP-2, IGF-I, or BD-1), which may be useful for early diagnosis and monitoring for SP patients. In addition, some inflammation-associated proteins (e.g., IGF-I sR, IGF-II, LBP, and LECT2) had similar alterations in ALI/ARDS and SP patients, which will be regarded as an index of evaluation for patients with inflammation, such as routine blood test. Our data from the present study also indicate that the activated cell migration and immune response occurred in SP-ARDS patients, including the gene cluster of CC chemokine receptor, monocyte chemotactic protein, cell migration, or immune response, biological process of immune response, monocyte chemotaxis, inflammatory response, or chemotaxis, and molecule function of chemokine receptor activity, chemoattractant activity, cytokine activity, and chemokine activity.

Our data demonstrate that elevated levels of certain inflammatory mediators during SP-ARDS may be potential risk factors for the initiation of lung cancer. IL-6 plays an essential role and has the specificity to regulate the nature of inflammation in lung cancer microenvironment and has tumor-promoting actions (Ochoa et al. 2011). Clinical findings demonstrated that levels of IL-6 increased in the circulation and regional fluids were associated with poor prognosis of patients with malignant diseases (Uskudar Teke et al. 2015), and the antibody against IL-6 siltuximab could inhibit the phosphorylation of STAT3 tyrosine and cell growth in NSCLC (Song et al. 2011). Results from our data mining furthermore found that the up-expression of IL-6 could predict poor prognosis predictors in NSCLC and that IL-6 is also involved in the signal pathway of cell proliferation (Supplement Fig. 1). To better explore the potential association and interaction mechanisms between IL-6 and EMT in lung cancer cells, we stimulated A549 with IL-6 in vitro cell culture. Our data initially demonstrated that the expression of IL-6 is associated with EMT. It indicates that IL-6 may also regulate the process of EMT during the progression of NSCLC, by which cells change from a highly polarized epithelial phenotype with intact cell–cell junctions to a migratory mesenchymal phenotype to promote tumor cell metastasis (Sullivan et al. 2009). Molecular mechanisms by which selected disease-specific biomarkers may contribute to the development of ARDS as well as tumorigenesis of lung cancer mainly include alterations of cell motility (CXCR3, Supplement Fig. 2), cytokine production, cell adhesion or differentiation (IL-5, IL-5 R alpha, Supplement Fig. 3), survival and migration (CXCL16, Supplement Fig. 4), metabolism (leptin or orexin B, Supplement Fig. 5 or 6), or cell proliferation and adhesion (NOV/CCN3 or IGFBP-4, Supplement Fig. 7 or 8). Those factors are involved in the multi-processes of lung inflammation, injury, or tumorigenesis, e.g., IL-22 BP in Supplement Fig. 9 or glypican in Supplement Fig. 10, and associated with the inflammation (Supplement Fig. 11).

To better characterize disease-specific biomarkers, it needs to even test a large number of potential protein biomarkers. The disease specificity of those multiple cytokines requires the comparison among many lung diseases, particularly like ARDS that results from a complex process of initiation and progression of inflammation network. The present study specially focused on dynamic alterations of systemic biomarkers in patients with SP-ARDS. Simultaneous detection of multiple cytokines as a panel will provide a more powerful tool to quantifiably measure cytokines in different stages of ARDS, like in other diseases (Chen et al. 2012a). However, because of the limitations in available sample volume and cost, it is difficult to obtain results of many proteins from ELISA measurement. Additionally, the challenges were also encountered during the identification and validation, such as large variation of results, concentrations of peptides and proteins (Rose et al. 2004), or the specificity of serum proteins (Chen et al. 2010). Of many omics technologies, antibody microarray provides an opportunity to take insight into global protein expression profiles (Huang 2001) and may be used to detect multiple proteins from one sample in an accurate, reproducible, and rapid manner as a fast, high-throughput, and sensitive tool for identifying potential biomarkers and in detecting levels of plasma cytokines.

Another challenge is how to integrate genomic or proteomic data with clinical characteristics and directly benefit the patients (Marshall 2011). The existing score index, such as the BODE index (Celli et al. 2004), has been used to monitor and provide good predictive information in a clinical practice, while they are graded with only partial variables that are not adequate for high-throughput analysis. Thus, there is an urgent need to combine advanced proteomic biotechnologies, clinical proteomics, tissue imaging and profiling, and organ dysfunction score systems together, to improve the clinical outcomes of patients (Wang et al. 2006). We developed a new system DESS to translate clinical descriptive information on major symptoms, signs, biochemical analyses, and imaging into clinical informatics as the digital values, to integrate the clinical informatics with bioinformatics, and to correlate molecular measurement with clinical direct vision for physicians and shrink the distance between lab discovery and clinical condition. Our data from the present study indicates that the digital informatics of those selected clinical parameters was correlated with disease-specific biomarkers. We found that plasma levels of inflammatory mediators were watched with severities of clinical informatics at various stages of the disease and that the multi-factorial scoring system could be useful when assessing and monitoring outcomes in ARDS patients. However, there are important needs to test a large population of patients with SP or ARDS, even though it is a challenge due to the cost of microarray, the limit of patient number for the recruitment, and large amount of works on clinical informatics. It should be also pointed out that the amount measured in the present study is the part of inflammatory mediators. The microarray used in the present study provided the information on relative folds of changes, rather than exact amount of circulating proteins. The correlation in the density of the spots for a particular protein with disease status may not necessarily mean that protein is directly involved in the disease process. In addition, it would be more helpful if there is a mathematical model to correlate proteomic-based bioinformatics with clinical informatics, so bioinformatics can be interpreted into clinical prediction and monitoring with the computational assistance. The efficiency of DESS used in the present study for clinical bioinformatics needs to be furthermore evaluated in the future.

In conclusion, we explored the feasibility and reliability of a new protocol of disease-specific biomarker evaluation by integrating proteomic profiles of inflammatory mediators in different ARDS stages, with clinical informatics. We measured 507 plasma inflammatory mediators and found 13 ARDS-specific biomarker candidates in patients different from both healthy and SP patients. We also explored the correction between selected biomarkers with DESS scores of patients to understand potential link of proteomic profile with clinical findings. There is a need to validate the predictive capability of those mediators in a large population of patients and clarify their specificity to ARDS as compared with levels in other pulmonary diseases and develop a simple and practical method which could serve as an aid in clinical practice and in the context of ARDS research.

## Electronic supplementary material

Below is the link to the electronic supplementary material.

Table S1 The flow chart of the recruited patients (DOC 43 kb)

Table S2 Variables and point values used for new score system (history and signs) (DOC 50 kb)

Table S3 Variables and point values used for new score system (signs) (DOC 39 kb)

Table S4 Variables and point values used for new score system (laboratory tests and imaging) (DOC 66 kb)

Table S5 Correlation between inflammatory mediators and DESS variables of symptoms (only *P* < 0.05 were showed) (DOC 58 kb)

Table S6 Correlation between inflammatory mediators and DESS variables of signs (only *P* < 0.05 were showed) (DOC 47 kb)

Table S7 Correlation between inflammatory mediators and DESS variables of laboratory tests (only *P* < 0.05 were showed) (DOC 55 kb)

Table S8 Correlation between inflammatory mediators and DESS variables of laboratory tests and imaging (only *P* < 0.05 were showed) (DOC 47 kb)

Fig. 1 (GIF 199 kb)

High resolution image (TIF 281 kb)

Fig. 2 (GIF 235 kb)

High resolution image (TIF 334 kb)

Fig. 3 (GIF 231 kb)

High resolution image (TIF 337 kb)

Fig. 4 (GIF 266 kb)

High resolution image (TIF 404 kb)

Fig. 5 (GIF 222 kb)

High resolution image (TIF 304 kb)

Fig. 6 (GIF 264 kb)

High resolution image (TIF 417 kb)

Fig. 7 (GIF 222 kb)

High resolution image (TIF 294 kb)

Fig. 8 (GIF 234 kb)

High resolution image (TIF 341 kb)

Fig. 9 (GIF 204 kb)

High resolution image (TIF 309 kb)

Fig. 10 (GIF 161 kb)

High resolution image (TIF 261 kb)

Fig. 11 (GIF 240 kb)

High resolution image (TIF 391 kb)
