# Supplementary figures and images for: Disease-specific dynamic biomarkers selected by integrating inflammatory mediators with clinical informatics in ARDS patients with severe pneumonia
Source: Cell Biol Toxicol. 2016 Apr 19;32:169–84. doi: 10.1007/s10565-016-9322-4 (PMC4882347; doi:10.1007/s10565-016-9322-4)

Supplement Table 1. The flow-chart of the recruited patients.


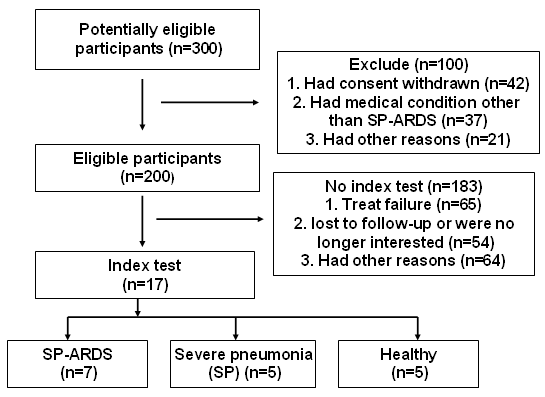

Supplement: Supplementary file 1 — The flow chart of the recruited patients (DOC 43 kb) [file 10565_2016_9322_MOESM1_ESM.doc]

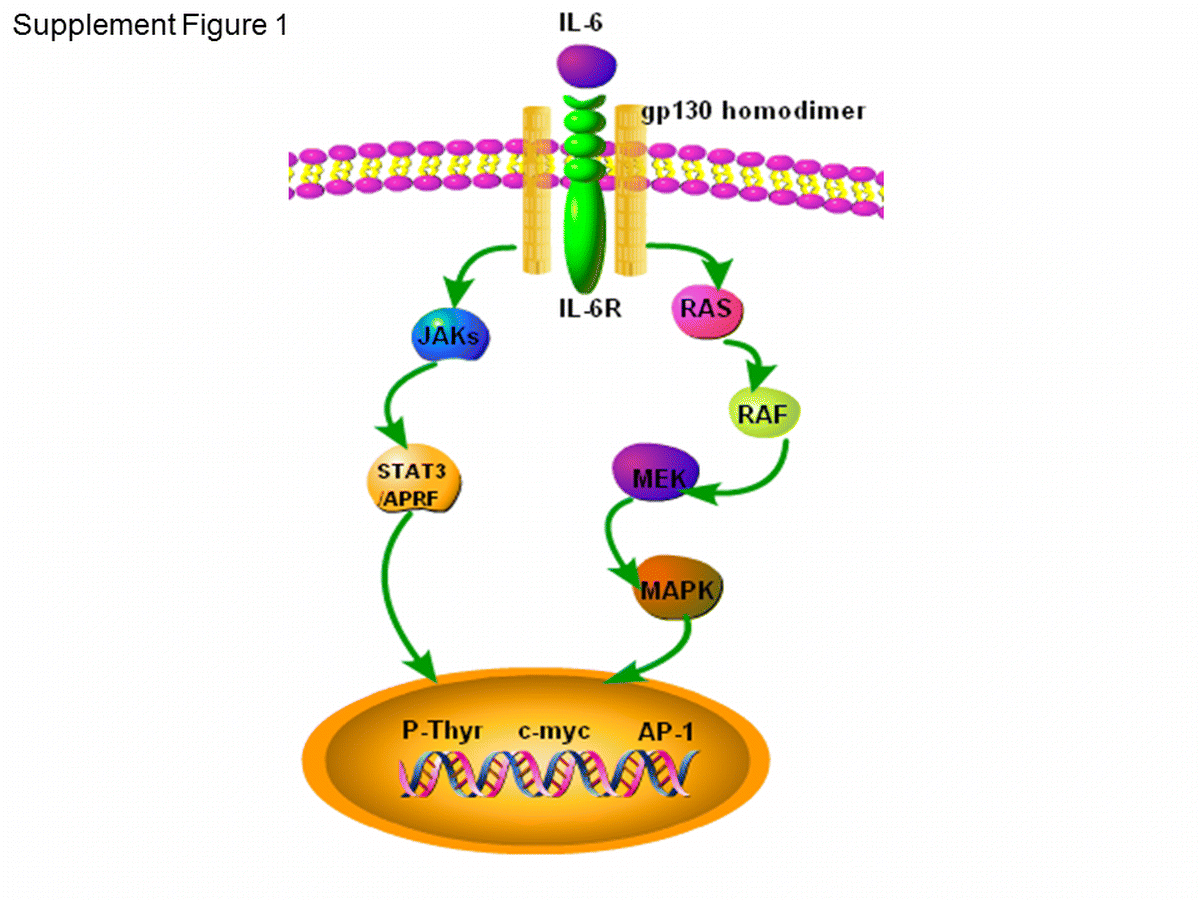

Supplement: Supplementary file 9 — (GIF 199 kb) [file 10565_2016_9322_Fig7_ESM.gif]

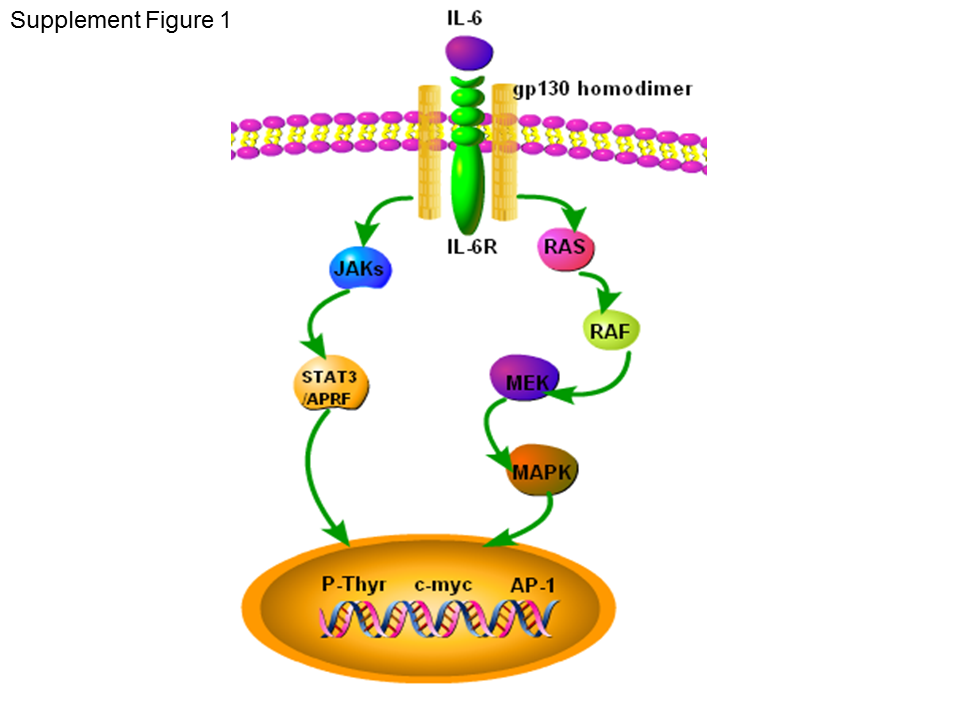

Supplement: Supplementary file 10 — High resolution image (TIF 281 kb) [file 10565_2016_9322_MOESM9_ESM.tif]

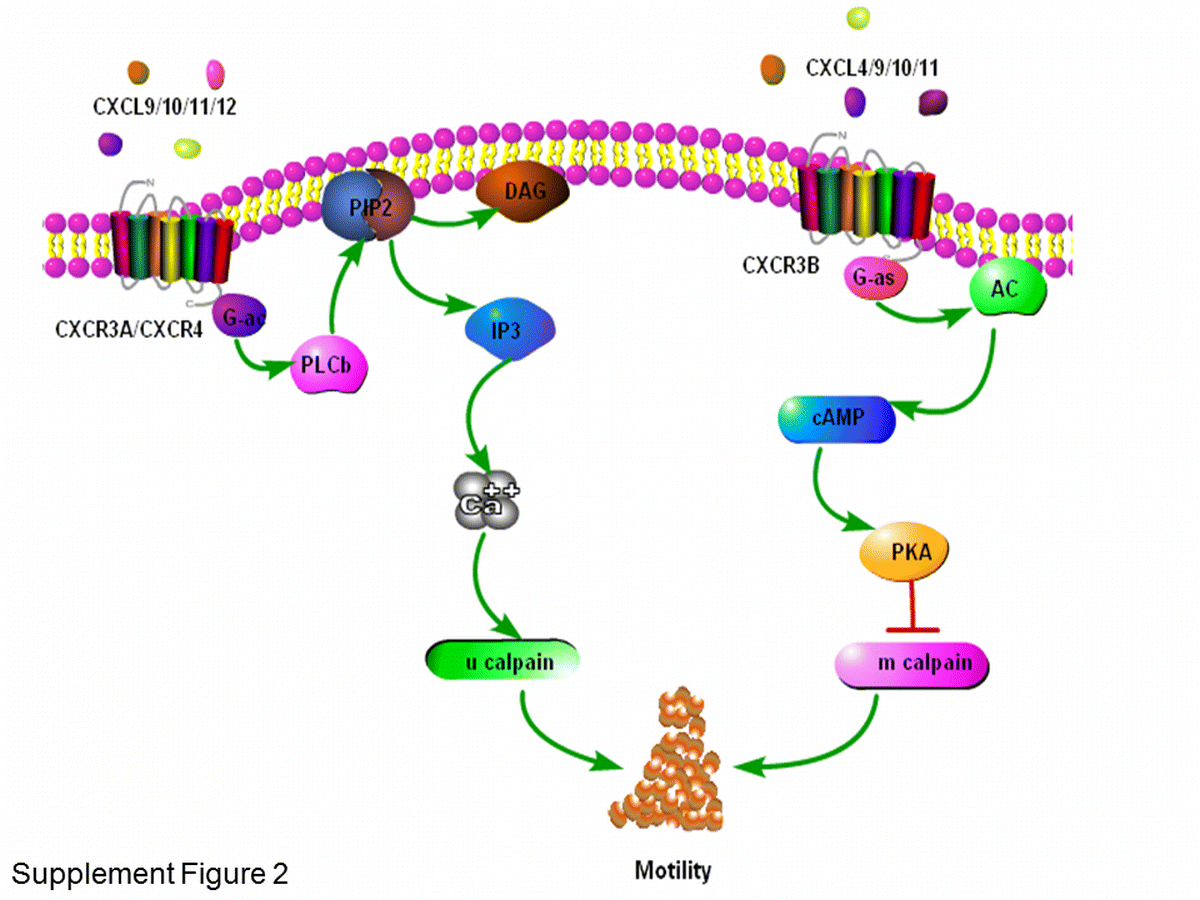

Supplement: Supplementary file 11 — (GIF 235 kb) [file 10565_2016_9322_Fig8_ESM.gif]

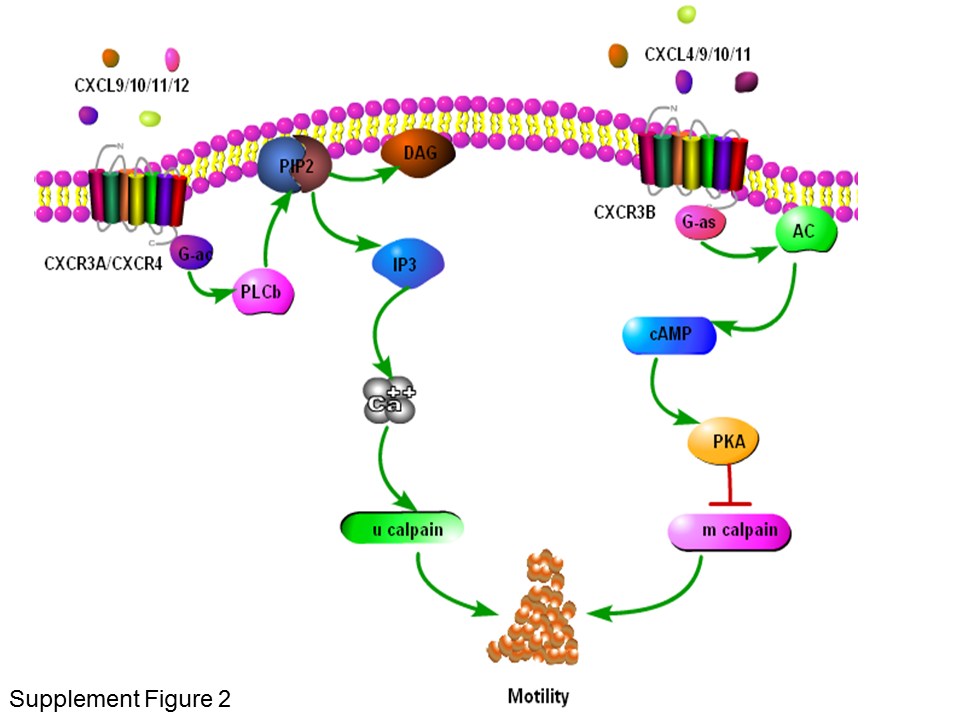

Supplement: Supplementary file 12 — High resolution image (TIF 334 kb) [file 10565_2016_9322_MOESM10_ESM.tif]

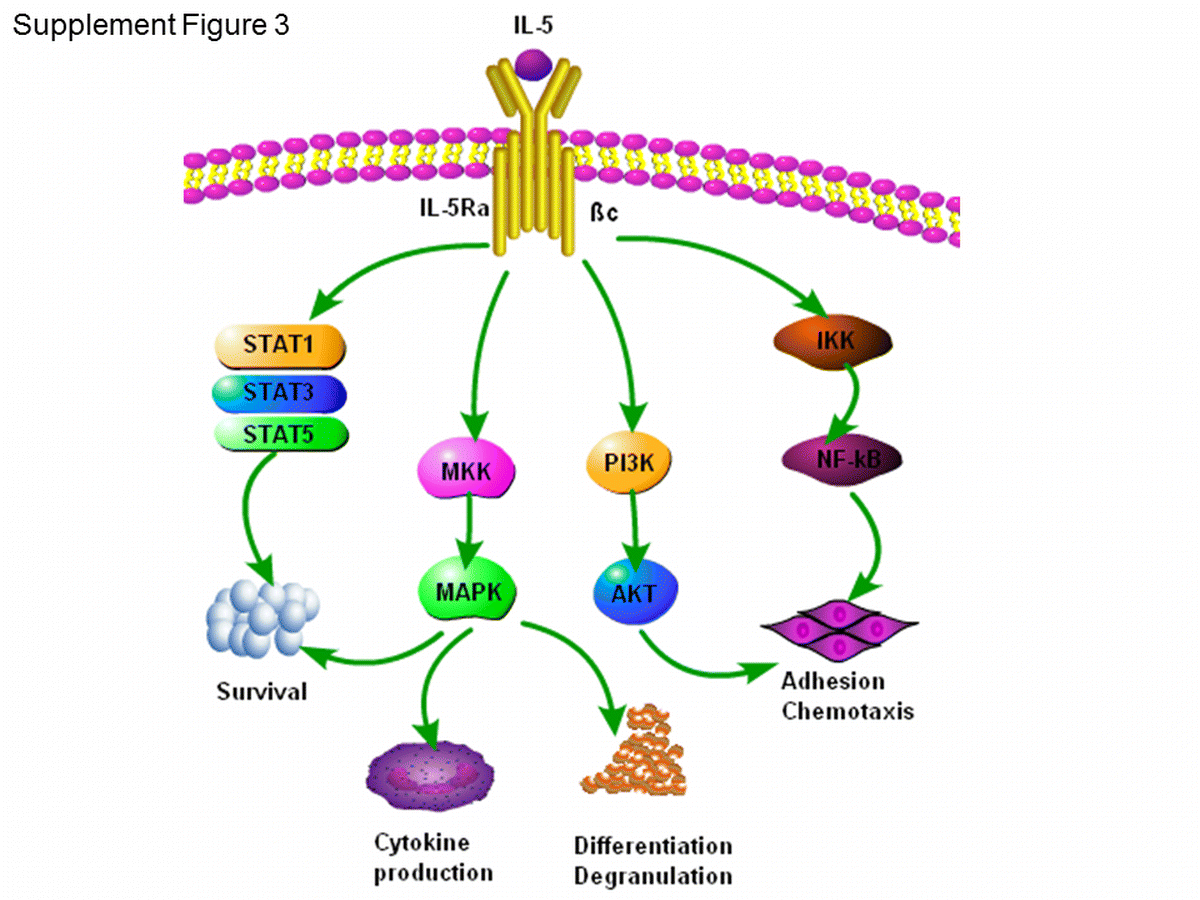

Supplement: Supplementary file 13 — (GIF 231 kb) [file 10565_2016_9322_Fig9_ESM.gif]

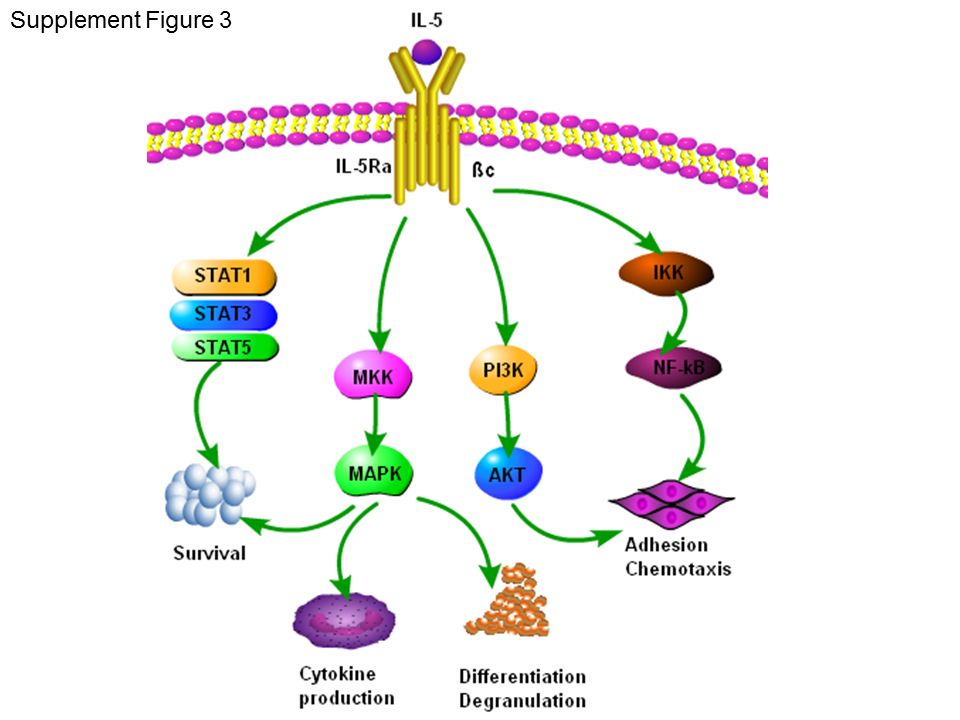

Supplement: Supplementary file 14 — High resolution image (TIF 337 kb) [file 10565_2016_9322_MOESM11_ESM.tif]

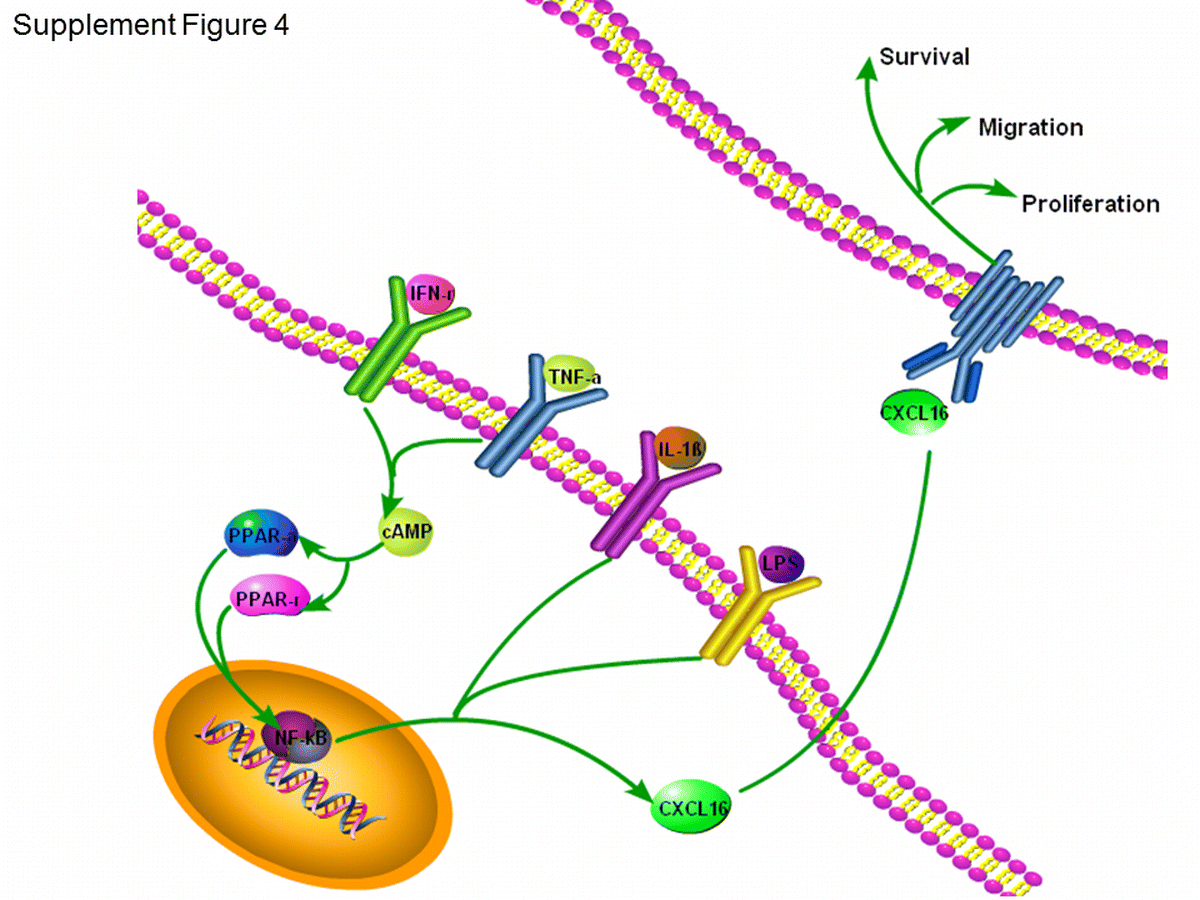

Supplement: Supplementary file 15 — (GIF 266 kb) [file 10565_2016_9322_Fig10_ESM.gif]

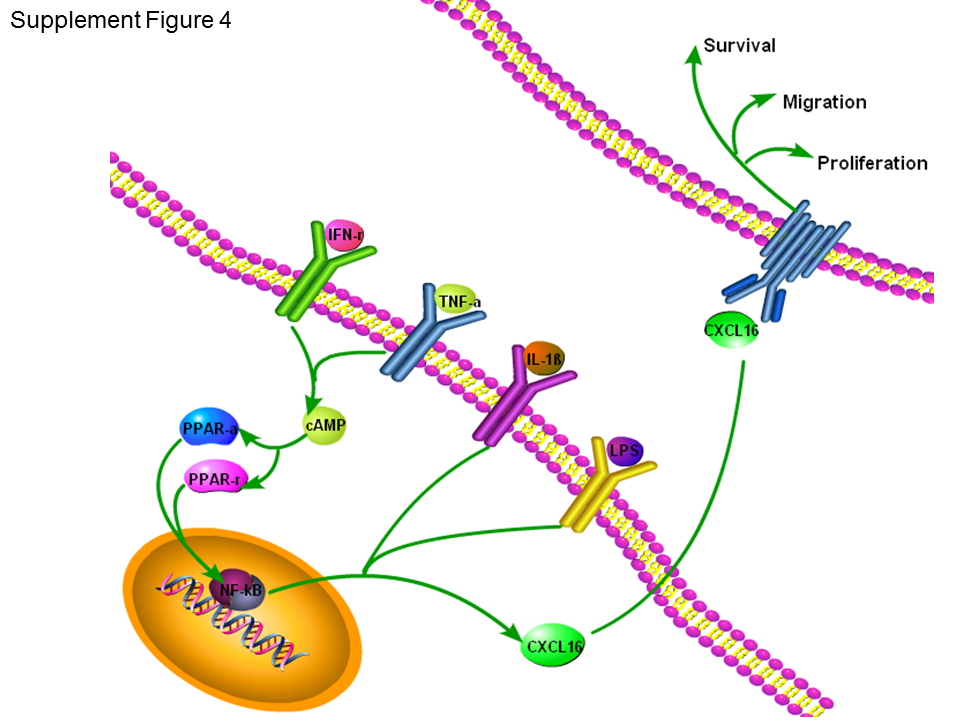

Supplement: Supplementary file 16 — High resolution image (TIF 404 kb) [file 10565_2016_9322_MOESM12_ESM.tif]

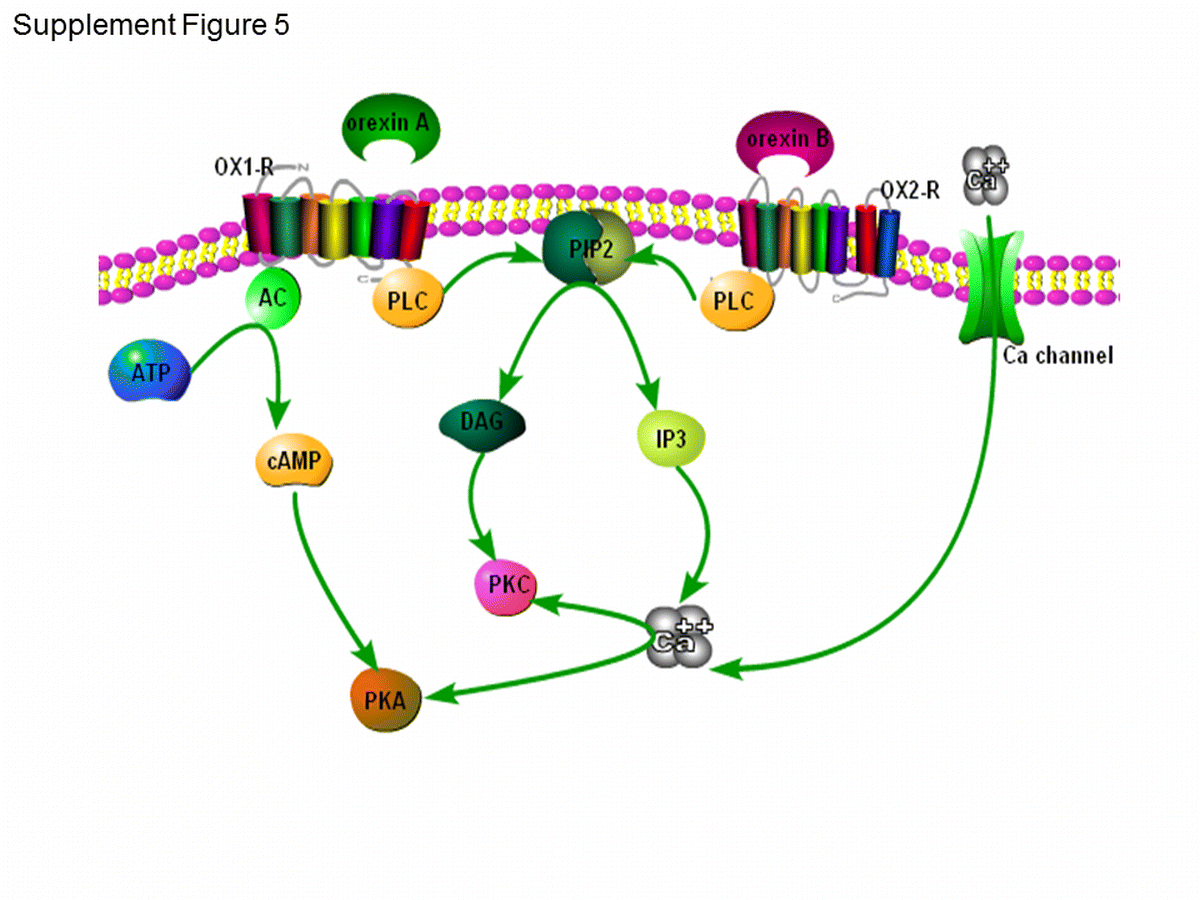

Supplement: Supplementary file 17 — (GIF 222 kb) [file 10565_2016_9322_Fig11_ESM.gif]

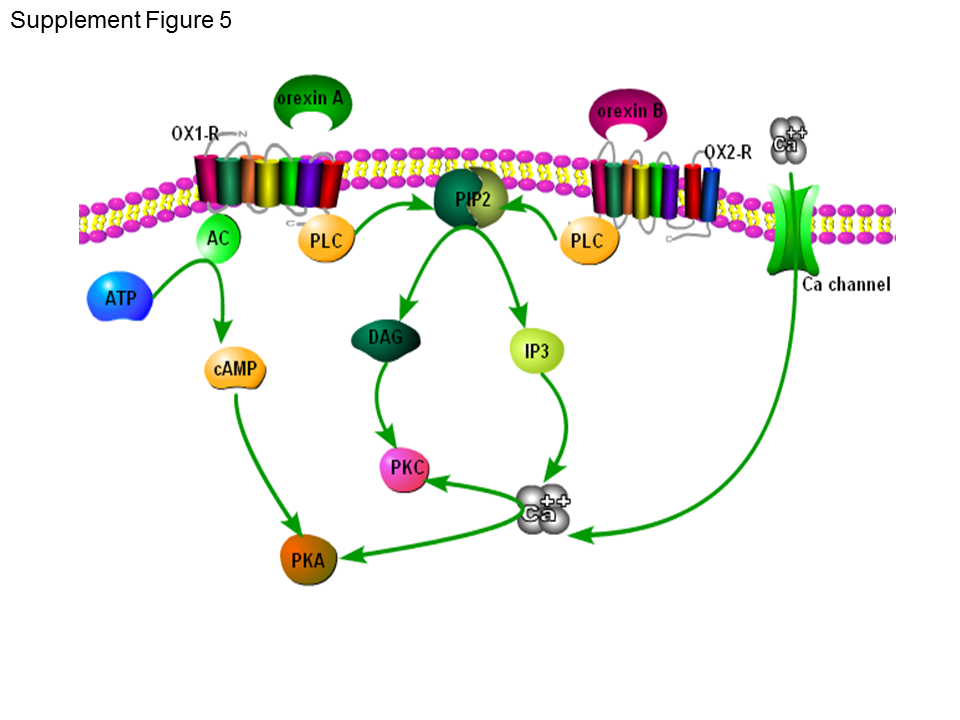

Supplement: Supplementary file 18 — High resolution image (TIF 304 kb) [file 10565_2016_9322_MOESM13_ESM.tif]

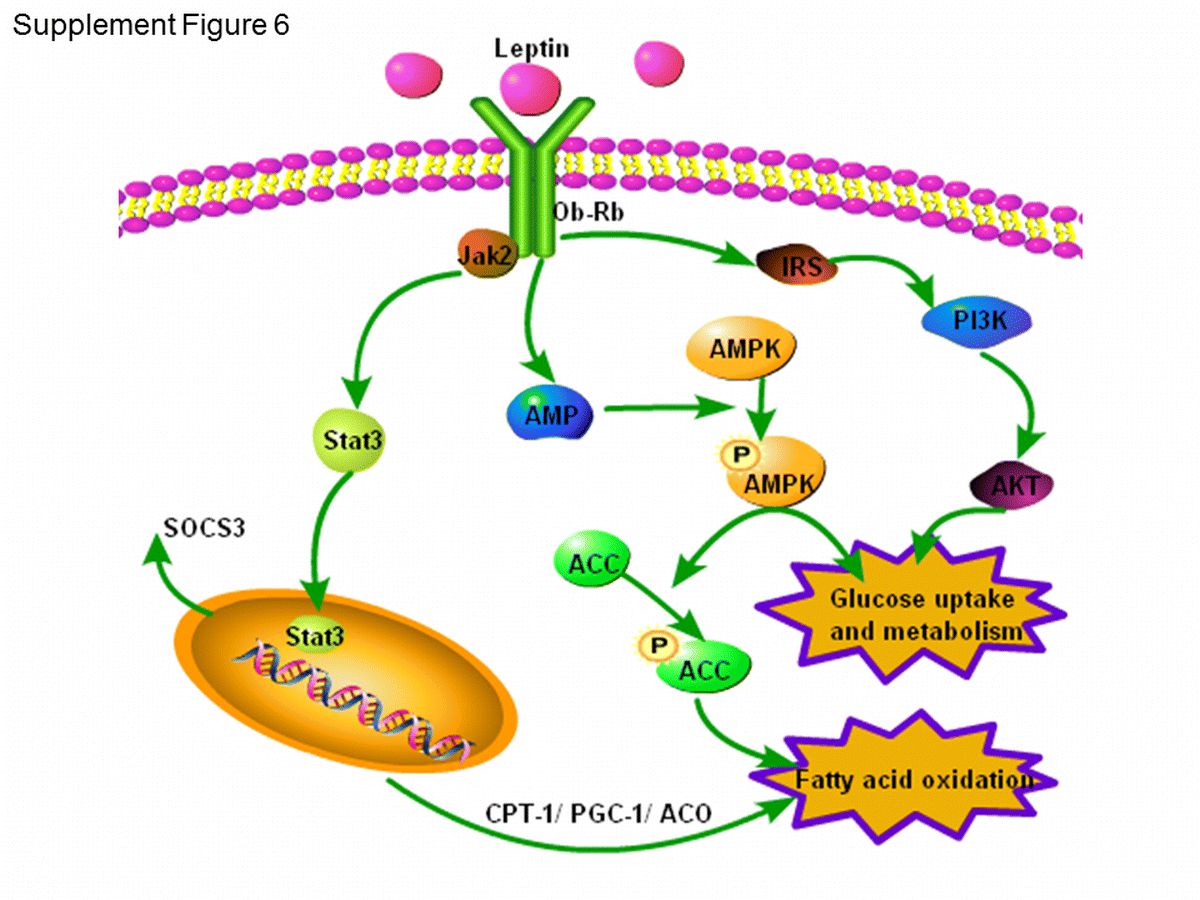

Supplement: Supplementary file 19 — (GIF 264 kb) [file 10565_2016_9322_Fig12_ESM.gif]

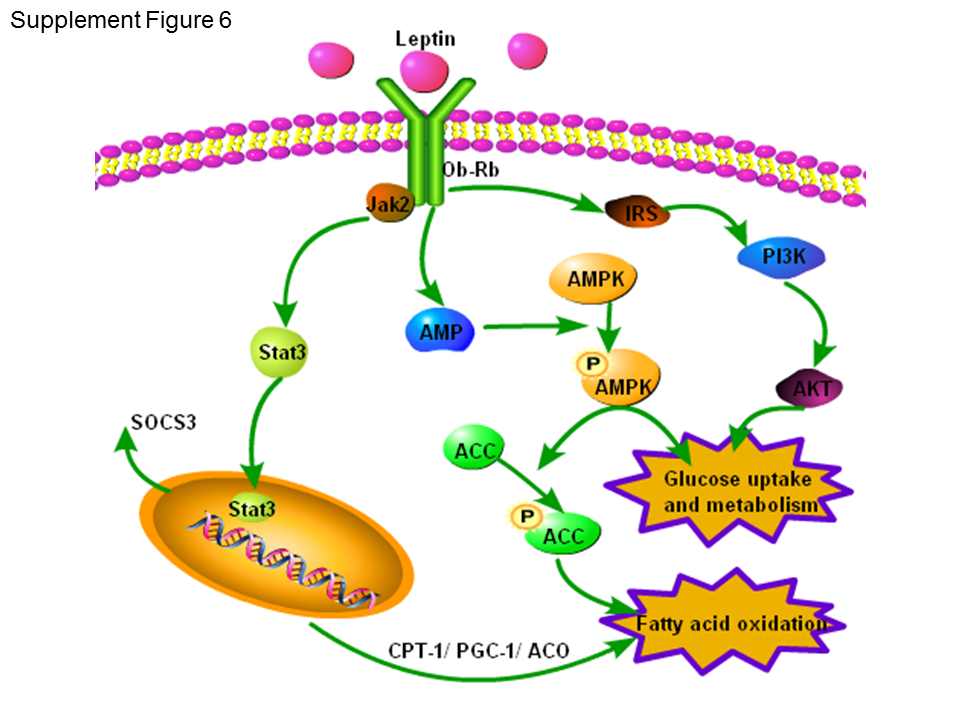

Supplement: Supplementary file 20 — High resolution image (TIF 417 kb) [file 10565_2016_9322_MOESM14_ESM.tif]

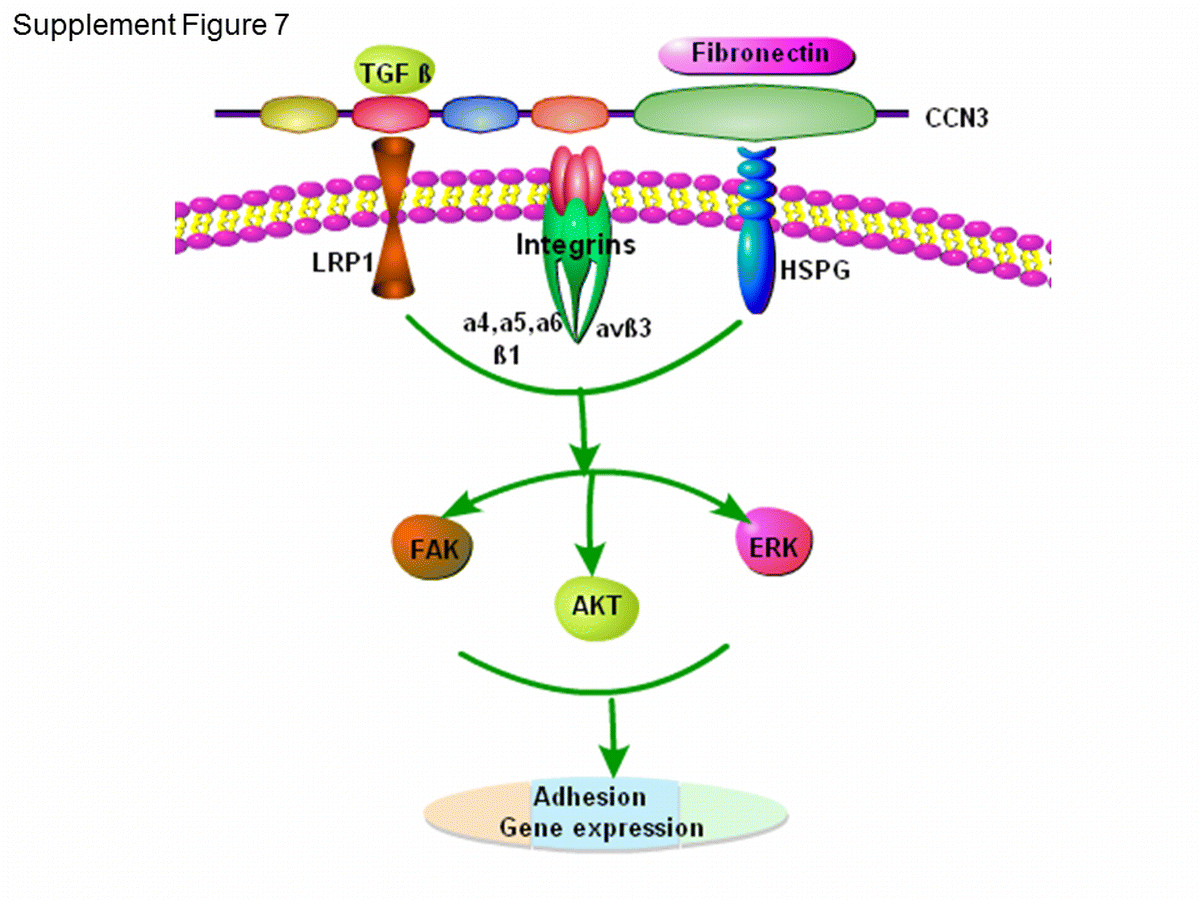

Supplement: Supplementary file 21 — (GIF 222 kb) [file 10565_2016_9322_Fig13_ESM.gif]

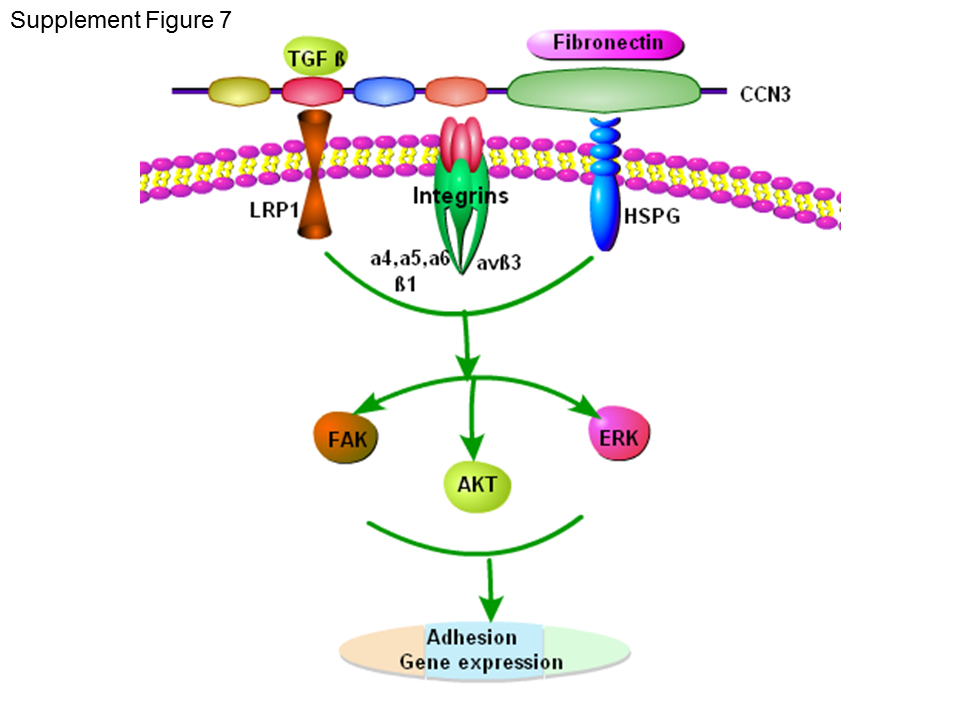

Supplement: Supplementary file 22 — High resolution image (TIF 294 kb) [file 10565_2016_9322_MOESM15_ESM.tif]

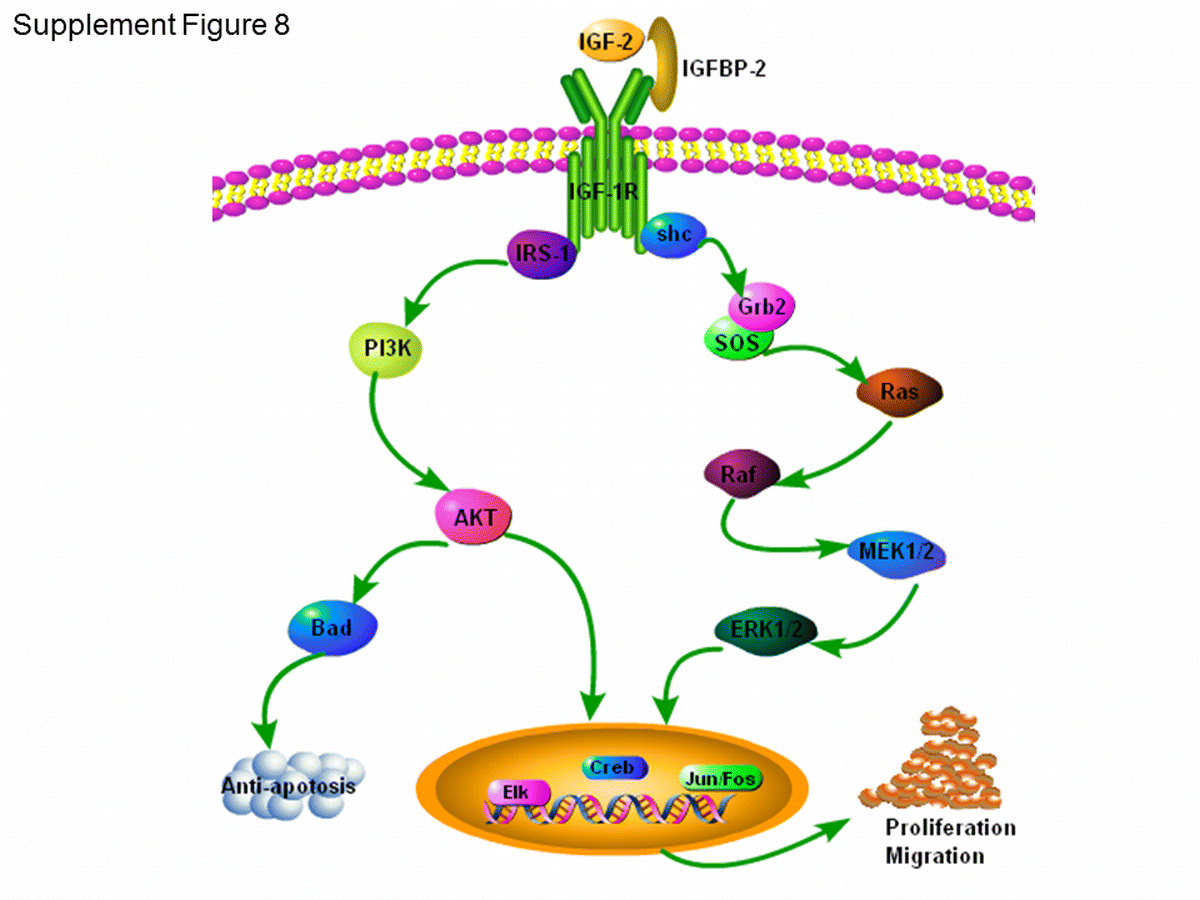

Supplement: Supplementary file 23 — (GIF 234 kb) [file 10565_2016_9322_Fig14_ESM.gif]

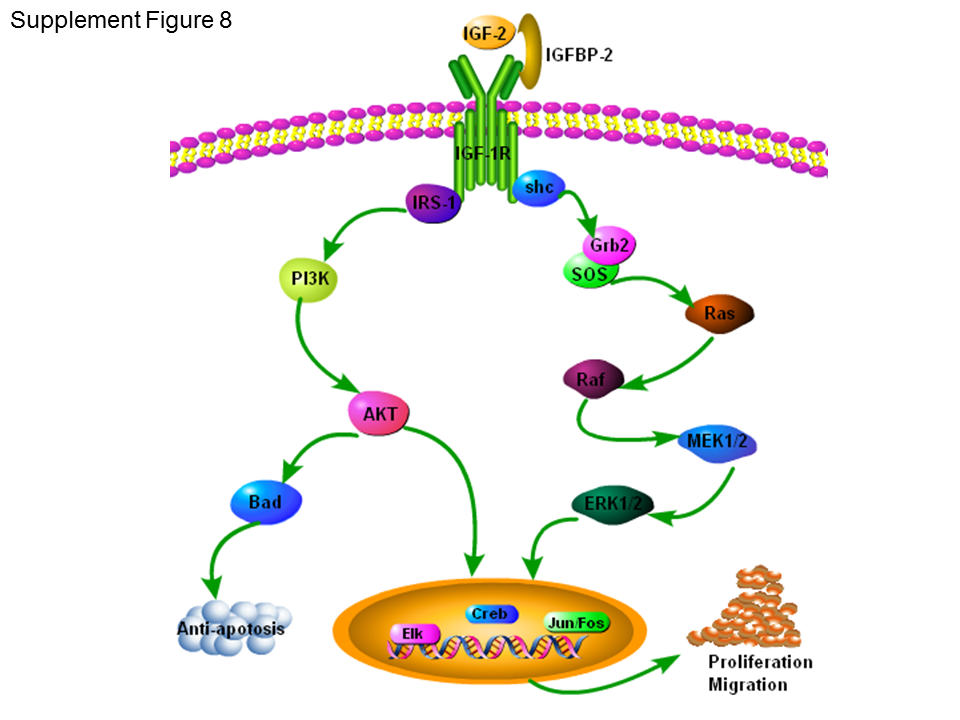

Supplement: Supplementary file 24 — High resolution image (TIF 341 kb) [file 10565_2016_9322_MOESM16_ESM.tif]

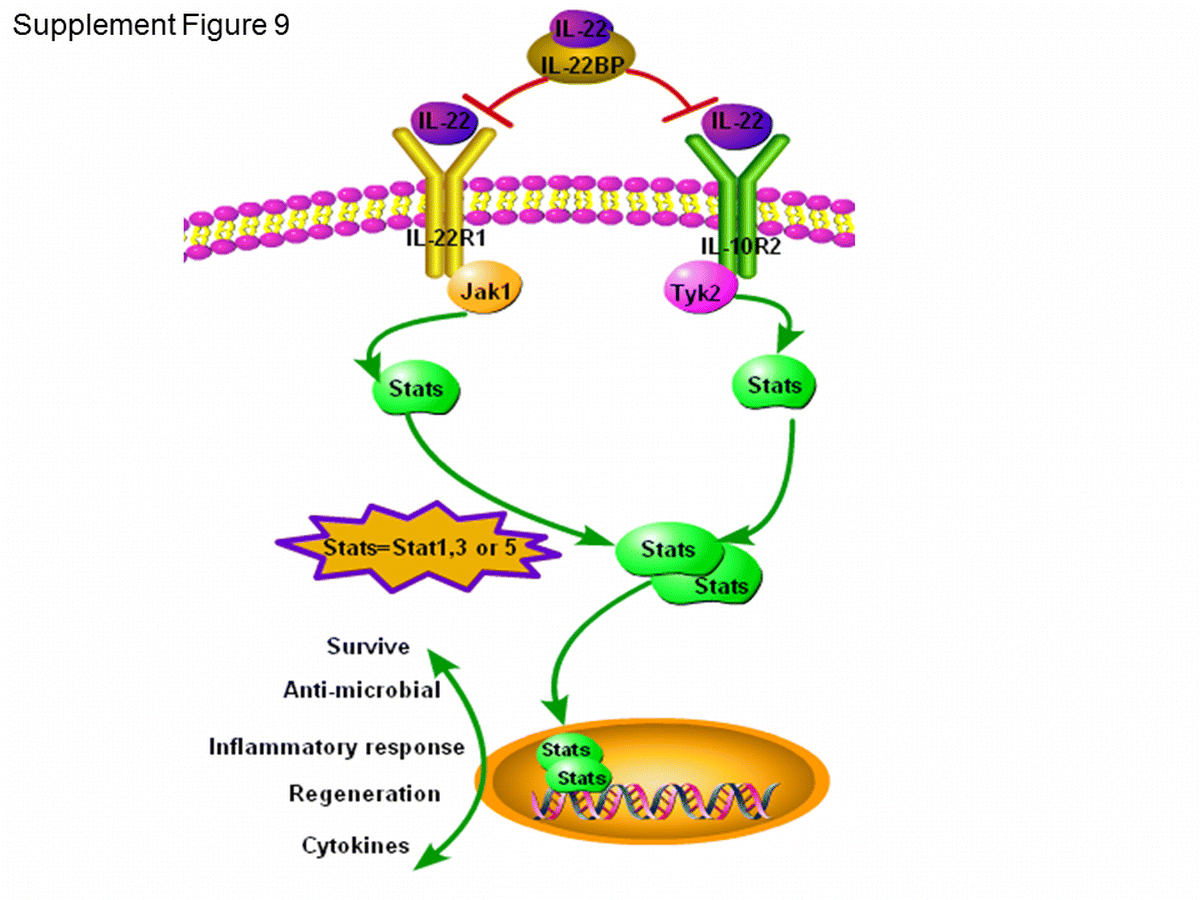

Supplement: Supplementary file 25 — (GIF 204 kb) [file 10565_2016_9322_Fig15_ESM.gif]

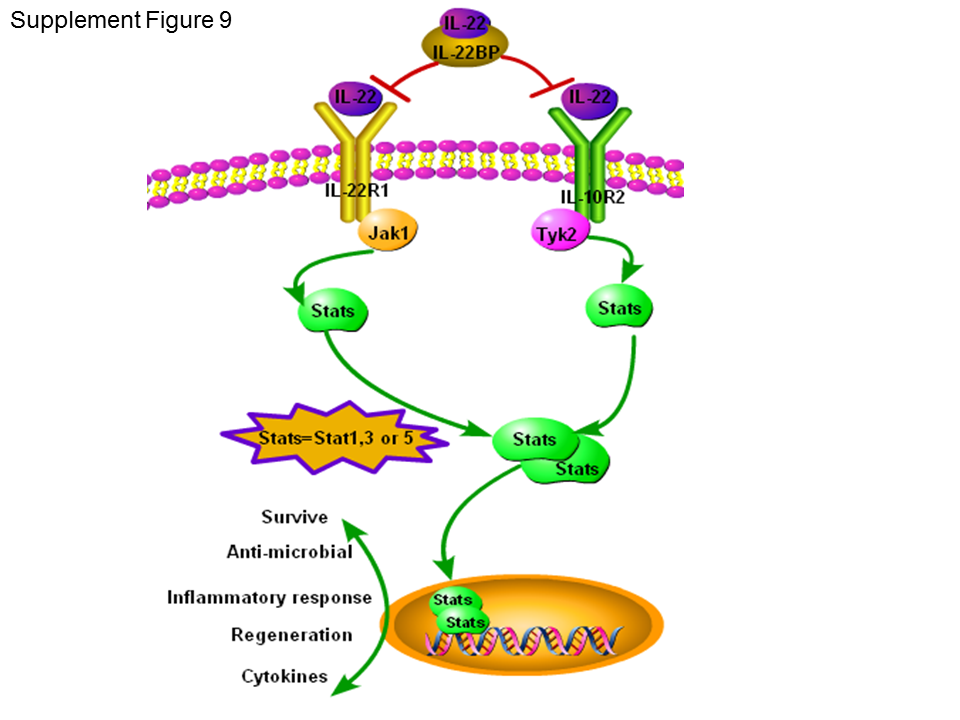

Supplement: Supplementary file 26 — High resolution image (TIF 309 kb) [file 10565_2016_9322_MOESM17_ESM.tif]

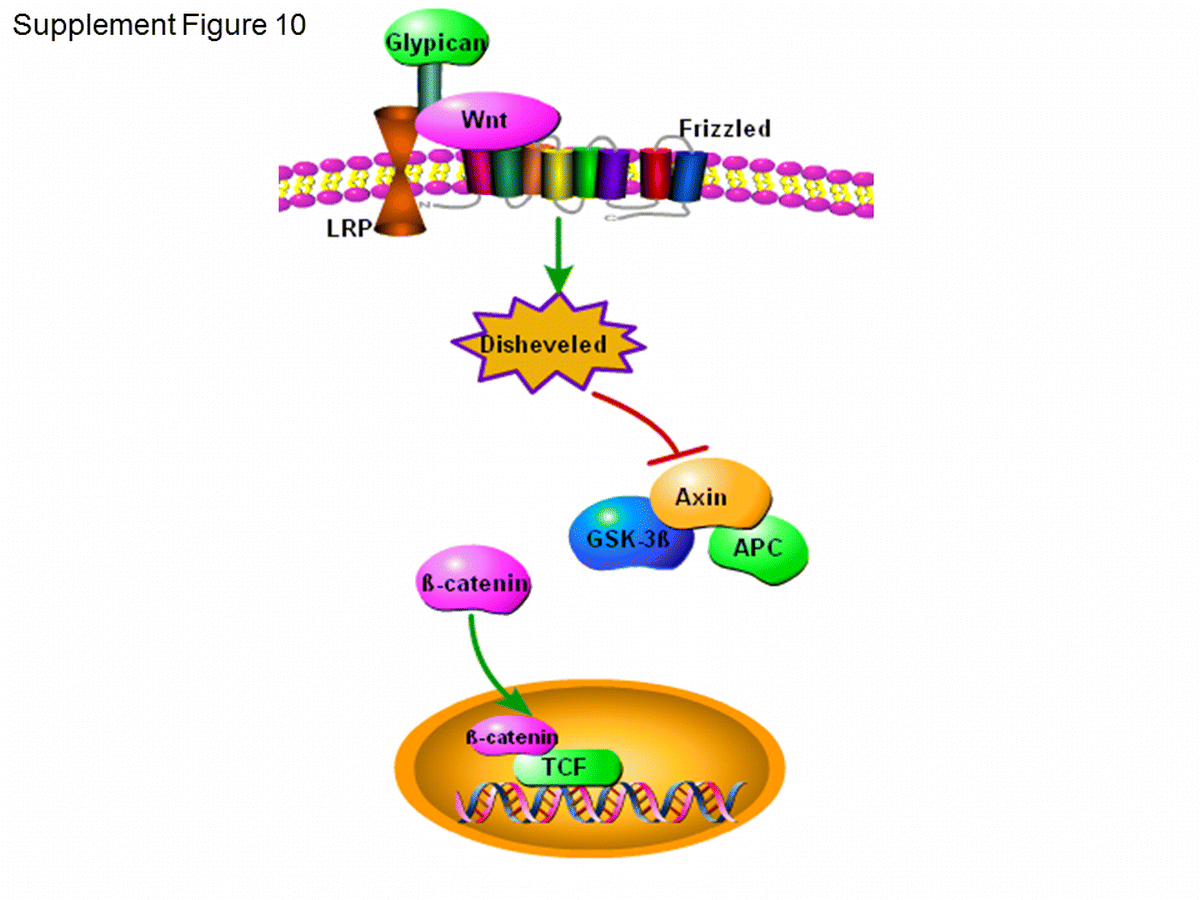

Supplement: Supplementary file 27 — (GIF 161 kb) [file 10565_2016_9322_Fig16_ESM.gif]

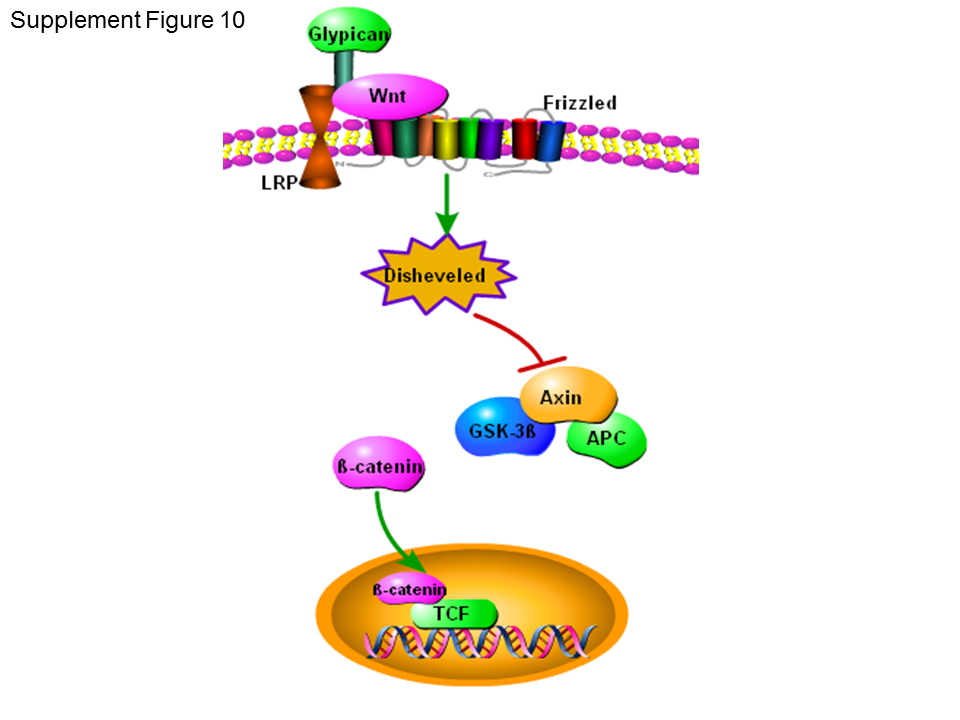

Supplement: Supplementary file 28 — High resolution image (TIF 261 kb) [file 10565_2016_9322_MOESM18_ESM.tif]

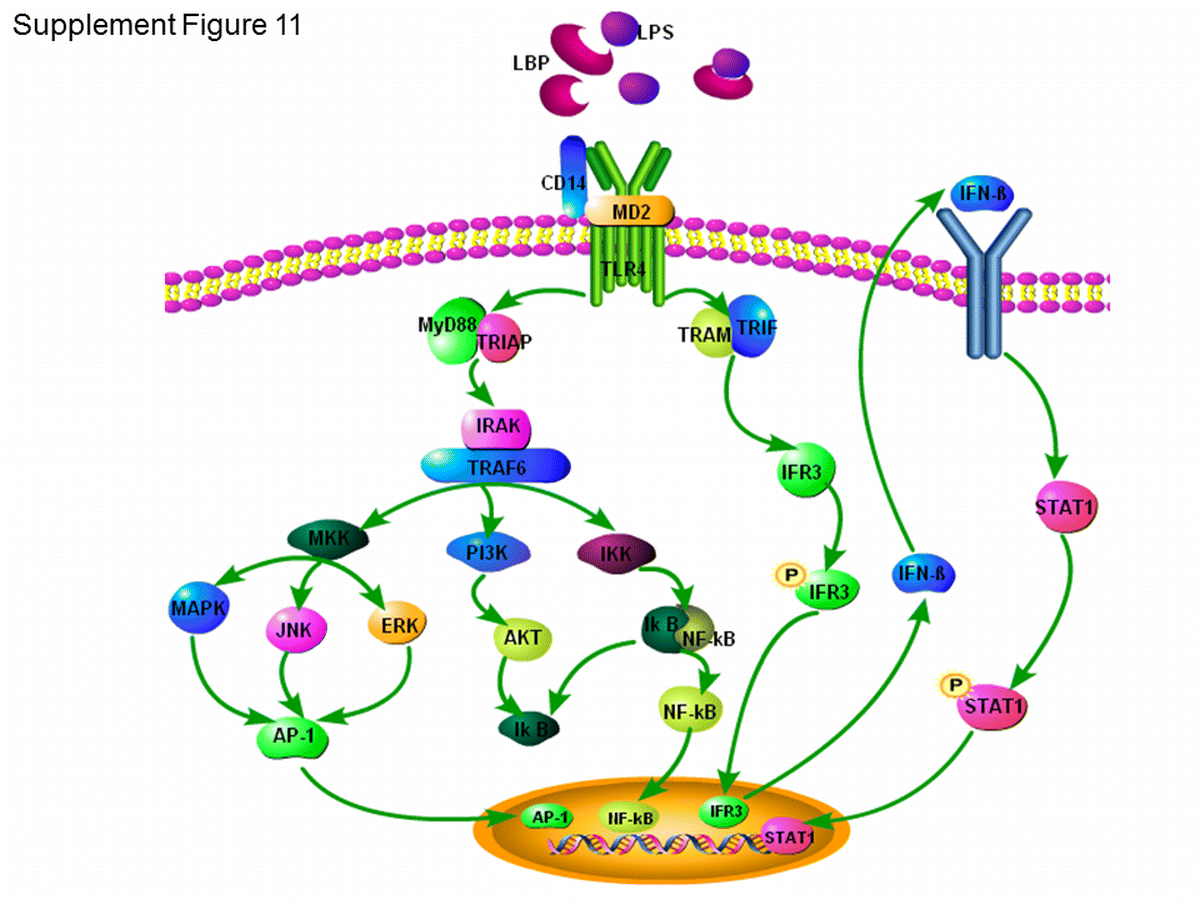

Supplement: Supplementary file 29 — (GIF 240 kb) [file 10565_2016_9322_Fig17_ESM.gif]

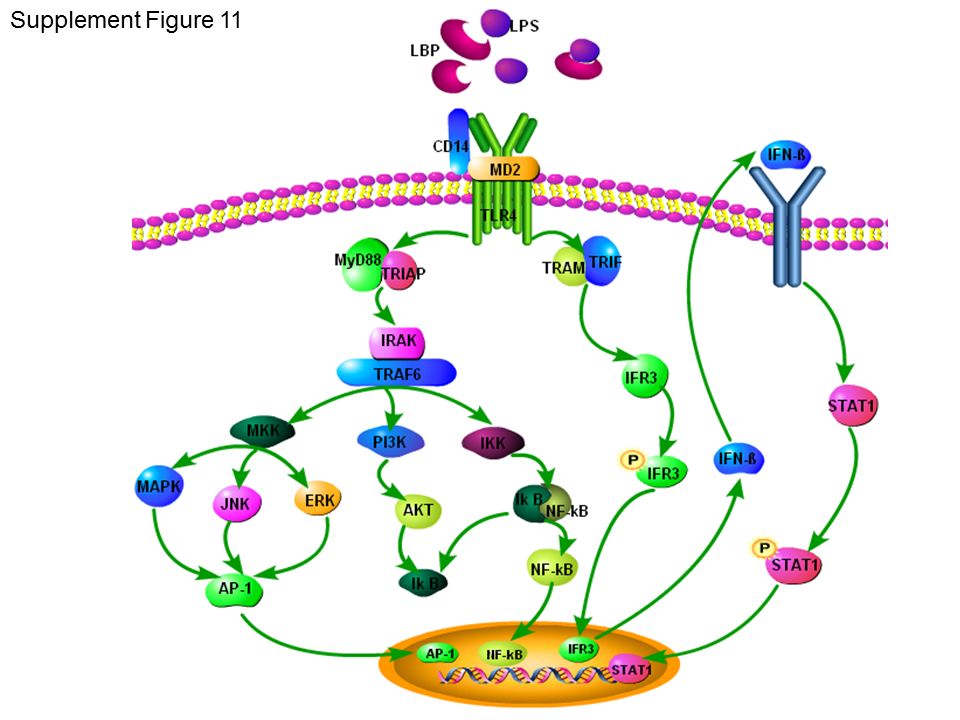

Supplement: Supplementary file 30 — High resolution image (TIF 391 kb) [file 10565_2016_9322_MOESM19_ESM.tif]
