# Supplementary material for: Disease-specific dynamic biomarkers selected by integrating inflammatory mediators with clinical informatics in ARDS patients with severe pneumonia
Source: Cell Biol Toxicol. 2016 Apr 19;32:169–84. doi: 10.1007/s10565-016-9322-4 (PMC4882347; doi:10.1007/s10565-016-9322-4)
Supplement: Supplementary file 2 — Variables and point values used for new score system (history and signs) (DOC 50 kb) [file 10565_2016_9322_MOESM2_ESM.doc]

Supplement Table 2. Variables and point values used for new score system (history and signs)

| **Variables** | **Points** | | | |
| --- | --- | --- | --- | --- |
|  | 0 | 1 | 2 | 4 |
| ***History*** |  |  |  |  |
| Cough severeness | No | <1wk | 1-2wks | >2wks |
| Sputum | No | White, and Small amount | White, relatively larger amount | Yellow |
| Chest pain | No | Under severe activity | Under daily activity | At rest |
| Short breathness | No | Only under severe activity | In daily activity | At rest |
| Limitation of activity | No | Mild | Marked | Severe |
| Orthopnea at night | No |  |  | Yes |
| Edema of lower limbs | No |  |  | Yes |
| Chill | No |  |  | Yes |
| Fever(℃) | No | 37.3-38 | 38.1-39 | >39 |
| Duration of fever | No | <1wk | 1-2wks | >2wks |
| Appetite | Good | Semi-liquid diet | Liquid diet | Absolute diet |
| Hymoptysis | No | Small | Medium | large |
| Stool and urine | Normal |  |  | Abnormal |
| Consciousness | Conscious | Hypersomia | Confusion | Coma |
| Hypertension | No | <5years | 5-10years | >10years |
| Diabetes mellitus | No | <5years | 5-10years | >10years |
| Chronic obstructive pulmonary disease(COPD) | No | <10years | 10-20years | >20years |
