# Supplementary material for: Disease-specific dynamic biomarkers selected by integrating inflammatory mediators with clinical informatics in ARDS patients with severe pneumonia
Source: Cell Biol Toxicol. 2016 Apr 19;32:169–84. doi: 10.1007/s10565-016-9322-4 (PMC4882347; doi:10.1007/s10565-016-9322-4)
Supplement: Supplementary file 3 — Variables and point values used for new score system (signs) (DOC 39 kb) [file 10565_2016_9322_MOESM3_ESM.doc]

Supplement Table 3. Variables and point values used for new score system (signs)

| **Variables** | **Points** | | | |
| --- | --- | --- | --- | --- |
|  | 0 | 1 | 2 | 4 |
| Temperature(℃) | ﹤37.3 | 37.3-38 | 38.1-39 | >39.1 |
| Heart rate(beat/minute) | 60-100 |  |  | ＞100,or<60,or with any kind of arrhythmia |
| Respiratory rate(/minute) | 16-18 | 19-20,or12-15 | 21-24,or 8-11 | >24,or<8 |
| Blood pressure(mmHg) | <140/90 | Diastolic:140-159or Systolic 90-99 | Diastolic:160-179or Systolic 100-109 | Diastolic:≥180 or Systolic ≥110 |
| Nutrition | Good | Median |  | Poor or overweight |
| Enlargement of lymphnodes | No |  |  | Yes |
| Three depression sign | No |  |  | Yes |
| Barrel chest | No |  |  | Yes |
| Chest palpitation | Negative |  |  | Positive signs |
| Chest percussion | Negative |  |  | Positive signs |
| Rales | No | Single side<1/3 area | Signle side1/3-1/2,or bilateral <1/3 | Single side>1/2,or bilateral>1/3 |
| Heart examination | Negative |  |  | Positive signs |
| Abdominal examination | Negative |  |  | Positive signs |
