# Supplementary material for: Disease-specific dynamic biomarkers selected by integrating inflammatory mediators with clinical informatics in ARDS patients with severe pneumonia
Source: Cell Biol Toxicol. 2016 Apr 19;32:169–84. doi: 10.1007/s10565-016-9322-4 (PMC4882347; doi:10.1007/s10565-016-9322-4)
Supplement: Supplementary file 4 — Variables and point values used for new score system (laboratory tests and imaging) (DOC 66 kb) [file 10565_2016_9322_MOESM4_ESM.doc]

Supplement Table 4. Variables and point values used for new score system (laboratory tests and imaging)

| **Variables** | **Points** | | | | |
| --- | --- | --- | --- | --- | --- |
|  | | ***0*** | ***1*** | ***2*** | ***4*** |
| ***Laboratory tests*** | |  |  |  |  |
| Hemoglobin(g/L) | | Male:120-160  Female:110-150 | 90-lower limit of normal | 60-90 | <60 |
| WBC(×10^9^/L) | | 4-10 | 1.5-4 | <1.5 | >10,or <1.5 |
| Neutrophil percentage(%) | | 50-70 |  |  | >70,or <50 |
| Platelet(×10^9^/L) | | 100-300 |  |  | >300,or<100 |
| Albumin(g/L) | | 35-55 |  | 28-35 | <28 |
| ALT(U/L) | | <30 |  |  | >30 |
| AST(U/L) | | <50 |  |  | >50 |
| ALP(U/L) | | Within normal range |  |  | Beyond |
| Gamma-GT | | Within normal range |  |  | Beyond |
| Bilirubin(μmol/L) | | <34.2 | 34.2-171 | 171-342 | >342 |
| Urea (mmol/L) | | 2.5-7.1 | 7.1-9 | 9-20 | >20 |
| Creatinine(μmol/L) | | 40-120 | 120-150 | 150-200 | >200 |
| Cholesterol(mmol/L) | | 3.1-5.9 | 5.9-7 | 7-8 | >8 |
| Triglyceride(mmol/L) | | 0.6-2.0 | 2.0-3.0 | 3.0-4.0 | >4.0 |
| HDL(mmol/L) | | 1.03-2.07 | 0.91-1.03 |  | <0.91 |
| LDL(mmol/L) | | <3.12 | 3.12-3.16 | 3.16-3.64 | >3.64 |
| Na(mmol/L) | | 135-145 | 146-155,or125-134 | 156-165,or 115-124 | >165,or <115 |
| K(mmol/L) | | 3.5-5.5 | 3-3.4 | 2.5-2.9 | >5.5,or<2.5 |
| Cl(mmol/L) | | 95-105 |  |  | <95,or>105 |
| Ca(mmol/L) | | 2.25-2.58 |  |  | >2.58,or<2.25 |
| P(mmol/L) | | 0.97-1.61 |  |  | >1.61,or<0.97 |
| Glycosylated hemoglobin,HbA1c(%) | | 4-6 | 6-8 | 8-9 | >9 |
| pH | | 7.35-7.45 |  |  | >7.45,or<7.35 |
| PaO2(mmHg) | | >90 | 60-90 | 40-60 | <40 |
| PaCO2(mmHg) | | 35-45 | 45-50 |  | >50 |
| SaO2(%) | | >90 | 80-90 | 60-80 | <60 |
| Increased numbers of Tumor marker | | 0 | 1-3 | 4-6 | >6 |
| C-reactive protein,CRP(mg/L) | | <10 | 10-30 | 30-90 | >90 |
| Prothrombin time prolonged(sec) | | 0 | Within 4sec | 4-6 | >6 |
| Fasting blood glucose(mmol/L) | | <5.8 | 5.8-7 |  | >7 |
| ***Lung Imaging*** | |  |  |  |  |
| Lung consolidation | | No | Single side<1/3 area | Single side1/3-1/2 | Single side>1/2,or bilateral |
| Enlargement of lymph nodes | | No |  |  | yes |
| Pleural effusion | | No | Single side<1/3 area | Single side1/3-1/2 | Single side>1/2,or bilateral |
| Emphysema | | No |  |  | Yes |
