# Supplementary material for: Disease-specific dynamic biomarkers selected by integrating inflammatory mediators with clinical informatics in ARDS patients with severe pneumonia
Source: Cell Biol Toxicol. 2016 Apr 19;32:169–84. doi: 10.1007/s10565-016-9322-4 (PMC4882347; doi:10.1007/s10565-016-9322-4)
Supplement: Supplementary file 5 — Correlation between inflammatory mediators and DESS variables of symptoms (only P < 0.05 were showed) (DOC 58 kb) [file 10565_2016_9322_MOESM5_ESM.doc]

Supplement table 5. Correlation between inflammatory mediators and DESS variables of symptoms (only p<0.05 were showed).

| **inflammatory mediators** | **Cough severness** | | **Sputum** | | **Chest pain** | | **Short breathness** | | **Limitation of activity** | | **Chill** | | **Fever** | | **Stool and urine** | | **Hypertension** | | **COPD years** | |
| --- | --- | --- | --- | --- | --- | --- | --- | --- | --- | --- | --- | --- | --- | --- | --- | --- | --- | --- | --- | --- |
|  | r | p | r | p | r | p | r | p | r | p | r | p | r | p | r | p | r | p | r | p |
| **BMP-15** | -.477 | .014 | -.624 | .019 |  |  |  |  |  |  |  |  |  |  | -.453 | .039 |  |  |  |  |
| **CXCL16** |  |  | -.574 | .005 |  |  |  |  |  |  |  |  | -.479 | .038 |  |  |  |  | -.695 | .003 |
| **CXCR3** | -.537 | .005 |  |  |  |  |  |  |  |  |  |  |  |  |  |  | .594 | .032 |  |  |
| **IL-6** |  |  | -.617 | .01 | -.576 | .05 |  |  |  |  | -.552 | .035 |  |  |  |  |  |  |  |  |
| **NOV / CCN3** |  |  |  |  |  |  | -.612 | .038 |  |  |  |  |  |  | -.516 | .031 |  |  |  |  |
| **Glypican 3** |  |  |  |  | -.572 | .021 |  |  |  |  |  |  | -.595 | .017 |  |  |  |  |  |  |
| **IGFBP-4** | -.613 | .024 | -.389 | .043 |  |  |  |  |  |  | -.415 | .034 |  |  |  |  |  |  |  |  |
| **IL-5** | .557 | .048 |  |  | -.519 | .038 |  |  |  |  |  |  |  |  |  |  |  |  |  |  |
| **IL-5 R alpha** |  |  | .478 | .037 |  |  |  |  | .397 | .045 |  |  |  |  |  |  |  |  |  |  |
| **IL-22 BP** |  |  |  |  |  |  | .462 | .041 |  |  |  |  | .559 | .004 |  |  |  |  |  |  |
| **Leptin (OB)** |  |  |  |  |  |  | .539 | .039 | .602 | .031 |  |  |  |  |  |  | .566 | .028 |  |  |
| **MIP-1d** | .611 | .003 |  |  |  |  |  |  |  |  |  |  | .444 | .033 |  |  |  |  |  |  |
| **Orexin B** |  |  |  |  |  |  |  |  | .514 | .036 |  |  |  |  | .412 | .049 |  |  |  |  |
