# Supplementary material for: Disease-specific dynamic biomarkers selected by integrating inflammatory mediators with clinical informatics in ARDS patients with severe pneumonia
Source: Cell Biol Toxicol. 2016 Apr 19;32:169–84. doi: 10.1007/s10565-016-9322-4 (PMC4882347; doi:10.1007/s10565-016-9322-4)
Supplement: Supplementary file 6 — Correlation between inflammatory mediators and DESS variables of signs (only P < 0.05 were showed) (DOC 47 kb) [file 10565_2016_9322_MOESM6_ESM.doc]

Supplement table 6. Correlation between inflammatory mediators and DESS variables of signs (only p<0.05 were showed).

| **inflammatory mediators** | **Temperature** | | **Heart rate** | | **Respiratory rate** | | **Blood pressure** | | **Nutrition** | | **Rales** | |
| --- | --- | --- | --- | --- | --- | --- | --- | --- | --- | --- | --- | --- |
|  | r | p | r | p | r | p | r | p | r | p | r | p |
| **BMP-15** | .602 | .042 |  |  |  |  |  |  |  |  | -.519 | .044 |
| **CXCL16** |  |  |  |  |  |  |  |  | -.514 | .034 |  |  |
| **CXCR3** |  |  | .512 | .027 |  |  |  |  |  |  |  |  |
| **IL-6** | -.520 | .019 |  |  | -.613 | .029 |  |  |  |  | -.666 | .029 |
| **NOV / CCN3** |  |  |  |  |  |  |  |  | -.538 | .047 |  |  |
| **Glypican 3** |  |  | .500 | .033 |  |  |  |  |  |  |  |  |
| **IGFBP-4** |  |  |  |  |  |  | -.457 | .049 |  |  |  |  |
| **IL-5** | -.574 | .002 |  |  |  |  |  |  |  |  |  |  |
| **IL-5 R alpha** |  |  |  |  |  |  |  |  | -.689 | .036 | -.722 | .009 |
| **IL-22 BP** |  |  |  |  | .449 | .023 |  |  |  |  |  |  |
| **Leptin (OB)** |  |  | -.517 | .027 |  |  |  |  |  |  |  |  |
| **MIP-1d** |  |  |  |  |  |  |  |  |  |  | -.614 | .044 |
| **Orexin B** |  |  |  |  | -.512 | .035 | -.661 | .028 |  |  |  |  |
