# Supplementary material for: Disease-specific dynamic biomarkers selected by integrating inflammatory mediators with clinical informatics in ARDS patients with severe pneumonia
Source: Cell Biol Toxicol. 2016 Apr 19;32:169–84. doi: 10.1007/s10565-016-9322-4 (PMC4882347; doi:10.1007/s10565-016-9322-4)
Supplement: Supplementary file 7 — Correlation between inflammatory mediators and DESS variables of laboratory tests (only P < 0.05 were showed) (DOC 55 kb) [file 10565_2016_9322_MOESM7_ESM.doc]

Supplement table 7. Correlation between inflammatory mediators and DESS variables of laboratory tests (only p<0.05 were showed).

| **inflammatory mediators** | **WBC** | | **Neutrophil%** | | **Albumin** | | **ALT(U/L)** | | **AST(U/L)** | | **Na(mmol/L)** | | **K(mmol/L)** | | **Cl(mmol/L)** | | **Glycosylated hemoglobin,HA1c** | |
| --- | --- | --- | --- | --- | --- | --- | --- | --- | --- | --- | --- | --- | --- | --- | --- | --- | --- | --- |
|  | r | p | r | p | r | p | r | p | r | p | r | p | r | p | r | p | r | p |
| **BMP-15** |  |  |  |  |  |  |  |  |  |  |  |  | -.619 | .031 |  |  |  |  |
| **CXCL16** |  |  |  |  |  |  |  |  | .527 | .018 |  |  |  |  |  |  |  |  |
| **CXCR3** | .513 | .021 | .483 | .027 |  |  |  |  |  |  |  |  |  |  |  |  |  |  |
| **IL-6** | -.472 | .013 |  |  |  |  |  |  |  |  |  |  |  |  | .517 | .046 |  |  |
| **NOV / CCN3** |  |  | -.381 | .034 |  |  | .610 | .041 |  |  |  |  | -.443 | .044 |  |  |  |  |
| **Glypican 3** |  |  |  |  |  |  | -.445 | .004 | .622 | .031 |  |  |  |  |  |  | -.428 | .006 |
| **IGFBP-4** |  |  |  |  |  |  |  |  |  |  | -.543 | .022 | .468 | .032 |  |  |  |  |
| **IL-5** | -.571 | .047 | .590 | .005 |  |  |  |  |  |  |  |  |  |  |  |  |  |  |
| **IL-5 R alpha** |  |  |  |  |  |  |  |  |  |  | -.613 | .039 |  |  |  |  |  |  |
| **IL-22 BP** |  |  |  |  |  |  |  |  | .475 | .043 |  |  |  |  |  |  |  |  |
| **Leptin (OB)** |  |  |  |  | .519 | .041 |  |  |  |  |  |  |  |  |  |  |  |  |
| **MIP-1d** |  |  |  |  |  |  |  |  | .376 | .039 |  |  |  |  |  |  |  |  |
| **Orexin B** | .494 | .023 |  |  |  |  |  |  |  |  |  |  |  |  |  |  |  |  |
