# Supplementary material for: Disease-specific dynamic biomarkers selected by integrating inflammatory mediators with clinical informatics in ARDS patients with severe pneumonia
Source: Cell Biol Toxicol. 2016 Apr 19;32:169–84. doi: 10.1007/s10565-016-9322-4 (PMC4882347; doi:10.1007/s10565-016-9322-4)
Supplement: Supplementary file 8 — Correlation between inflammatory mediators and DESS variables of laboratory tests and imaging (only P < 0.05 were showed) (DOC 47 kb) [file 10565_2016_9322_MOESM8_ESM.doc]

Supplement table 8. Correlation between inflammatory mediators and DESS variables of laboratory tests and imaging (only p<0.05 were showed).

| **inflammatory mediators** | **pH** | | **PaO2** | | **PaCO2** | | **SaO2** | | **CRP** | | **Emphysema in imaging** | |
| --- | --- | --- | --- | --- | --- | --- | --- | --- | --- | --- | --- | --- |
|  | r | p | r | p | r | p | r | p | r | p | r | p |
| **BMP-15** | -.575 | .015 |  |  |  |  |  |  |  |  |  |  |
| **CXCL16** |  |  |  |  | -.474 | .016 |  |  |  |  |  |  |
| **CXCR3** |  |  |  |  | -436 | .031 |  |  | .518 | .048 |  |  |
| **IL-6** |  |  |  |  |  |  | . |  | 480 | .032 |  |  |
| **NOV / CCN3** | .545 | .019 |  |  |  |  | -.359 | .047 |  |  |  |  |
| **Glypican 3** |  |  |  |  | -.414 | .021 |  |  |  |  |  |  |
| **IGFBP-4** |  |  |  |  |  |  | -.513 | .039 |  |  |  |  |
| **IL-5** |  |  |  |  | -.618 | .005 |  |  |  |  | .441 | .044 |
| **IL-5 R alpha** |  |  | -.358 | .037 |  |  |  |  |  |  |  |  |
| **IL-22 BP** |  |  |  |  | -.513 | .035 |  |  |  |  |  |  |
| **Leptin (OB)** |  |  |  |  |  |  |  |  |  |  | -.514 | .021 |
| **MIP-1d** |  |  | .411 | .043 |  |  |  |  |  |  |  |  |
| **Orexin B** |  |  |  |  |  |  |  |  | -.636 | .012 | -.424 | .029 |
